# In vitro toxicoproteomic analysis of A549 human lung epithelial cells exposed to urban air particulate matter and its water-soluble and insoluble fractions

**DOI:** 10.1186/s12989-017-0220-6

**Published:** 2017-10-02

**Authors:** Ngoc Q. Vuong, Dalibor Breznan, Patrick Goegan, Julie S. O’Brien, Andrew Williams, Subramanian Karthikeyan, Premkumari Kumarathasan, Renaud Vincent

**Affiliations:** 1 Inhalation Toxicology Laboratory, Environmental Health Science and Research Bureau, Health Canada, Ottawa, ON K1A 0K9 Canada; 2 Analytical Biochemistry and Proteomics, Environmental Health Science and Research Bureau, Health Canada, Ottawa, ON K1A 0K9 Canada; 3 Biostatistics Section, Population Studies Division, Environmental Health Science and Research Bureau, Health Canada, Ottawa, ON K1A 0K9 Canada; 4 Department of Biochemistry, Faculty of Science, University of Ottawa, Ottawa, ON K1H 8M5 Canada

**Keywords:** Particulate matter (PM), EHC-93, Soluble fraction, Insoluble fraction, A549, Cytotoxicity, Toxicoproteomics, Two-dimensional gel electrophoresis (2D–GE), Mass spectrometry (MS)

## Abstract

**Background:**

Toxicity of airborne particulate matter (PM) is difficult to assess because PM composition is complex and variable due to source contribution and atmospheric transformation. In this study, we used an in vitro toxicoproteomic approach to identify the toxicity mechanisms associated with different subfractions of Ottawa urban dust (EHC-93).

**Methods:**

A549 human lung epithelial cells were exposed to 0, 60, 140 and 200 μg/cm^2^ doses of EHC-93 (total), its insoluble and soluble fractions for 24 h. Multiple cytotoxicity assays and proteomic analyses were used to assess particle toxicity in the exposed cells.

**Results:**

The cytotoxicity data based on cellular ATP, BrdU incorporation and LDH leakage indicated that the insoluble, but not the soluble, fraction is responsible for the toxicity of EHC-93 in A549 cells. Two-dimensional gel electrophoresis results revealed that the expressions of 206 protein spots were significantly altered after particle exposures, where 154 were identified by MALDI-TOF-TOF-MS/MS. The results from cytotoxicity assays and proteomic analyses converged to a similar finding that the effects of the total and insoluble fraction may be alike, but their effects were distinguishable, and their effects were significantly different from the soluble fraction. Furthermore, the toxic potency of EHC-93 total is not equal to the sum of its insoluble and soluble fractions, implying inter-component interactions between insoluble and soluble materials resulting in synergistic or antagonistic cytotoxic effects. Pathway analysis based on the low toxicity dose (60 μg/cm^2^) indicated that the two subfractions can alter the expression of those proteins involved in pathways including cell death, cell proliferation and inflammatory response in a distinguishable manner. For example, the insoluble and soluble fractions differentially affected the secretion of pro-inflammatory cytokines such as MCP-1 and IL-8 and distinctly altered the expression of those proteins (e.g., TREM1, PDIA3 and ENO1) involved in an inflammatory response pathway in A549 cells.

**Conclusions:**

This study demonstrated the impact of different fractions of urban air particles constituted of various chemical species on different mechanistic pathways and thus on cytotoxicity effects. In vitro toxicoproteomics can be a valuable tool in mapping these differences in air pollutant exposure-related toxicity mechanisms.

**Electronic supplementary material:**

The online version of this article (10.1186/s12989-017-0220-6) contains supplementary material, which is available to authorized users.

## Background

Airborne particulate matter (PM) is a complex mixture of particles with a wide range of sizes and physicochemical properties. Inhalation of airborne PM is linked to the development or exacerbation of respiratory illnesses such as bronchitis [26, 42, 46], asthma [11, 27, 47] and lung cancer [28, 33, 44]; and it is also associated with decline in cognitive function [3, 21, 23, 54] and increased risk of developing diabetes mellitus [9, 36, 60] and cardiovascular disease [15, 20, 32, 41, 58]. A number of epidemiological studies have reported that there is an association between particle composition and health impacts of ambient air particles [5, 10, 30, 66]. However, composition of the respirable particles can vary in different geographical locations depending on the local sources of release [10]. Thus, identifying the drivers of toxic potency and determining their mechanism of effects in airborne PM should be important and useful in the development of regulatory measures to reduce the negative health effects of air pollution.

There are several approaches to the identification of toxic components of ambient air PM. Some studies examined or regressed the toxic effect of the total particles to its water-soluble and/or insoluble components [19, 45, 55, 65], whereas others investigated the effects of particles with defined aerodynamic size range (e.g., <10 μm (PM10), <2.5 μm (PM2.5) and/or <0.1 μm ultrafine particles) in vitro or in vivo [4, 18, 52]. The limitation to most studies assessing the toxicity of PM is the ability to collect sufficient materials for physical and chemical characterization of the particles, and for in vitro and in vivo toxicological investigations. Thus, it is rare to find a single report that could provide all the important details regarding the physicochemical properties, relative cytotoxicities and mechanisms of particle toxicity of the total PM and its constituent components or sub-fractions.

In 1993, a large quantity of ambient air particles from the Environmental Health Centre in Ottawa (EHC-93) was collected to serve the purpose of a reference outdoor urban dust sample to use in different toxicological studies [56, 57]. Since then, EHC-93 has been used extensively in numerous in vivo and in vitro studies. EHC-93 has been partially characterized for the presence of various particle components such as endotoxin, polycyclic aromatic hydrocarbons and metal contents in its total particles [8, 57]. The potency of EHC-93 in causing oxidative/nitrative stress, inflammation and cardiovascular stress in animals has been well documented [2, 7, 24, 51, 56, 58]. EHC-93 was also reported to alter the expression of several genes and cytokines in animals and cells in the respiratory tract [8, 12, 14, 39, 51, 53]. However, a detailed proteomic investigation to assess the molecular mechanisms delineating the toxic effects of EHC-93 as a whole (total) or separated fractions (i.e., insoluble and soluble) has not been conducted. In our recent studies, we demonstrated that in vitro toxicoproteomics is an approach that is capable of distinguishing the pathways associated with cytotoxic effects of respirable particles that are different in physicochemical properties such as carbon black and titanium dioxide [62, 63]. Furthermore, we also showed that our in vitro toxicoproteomic approach was capable of differentiating the effects of particles that were identical in chemical formula (SiO_2_) but differed in physical properties such as cristobalite and α-quartz [61]. In this study, we used in vitro toxicoproteomics to dissect the effects of insoluble and soluble components of EHC-93 on A549 human lung epithelial cells. The results from this study showed that cytotoxicity assays, cytokine assays and proteomic analyses (based on two-dimensional gel electrophoresis and mass spectrometry) can differentiate the subtle differences in toxicity between EHC-93 total and its insoluble fraction as well as the drastic difference in toxicity between the soluble fraction and the total or insoluble fraction in A549 cells. To our knowledge, this is the first study that is able to provide extensive details on the physicochemical characteristics of an urban air PM and its sub-fractions, followed by comparing their cytotoxic potencies with multiple assays and comparing their associated cellular mechanisms of effects with proteomic analyses.

## Methods

### Materials

Culture flasks (T-25 and T-75), 96-well plates and plastic cell scraper were obtained from Corning Inc. (Corning, NY, USA). Dulbecco’s Modified Eagle’s Medium (DMEM) and fetal bovine serum (FBS) were purchased from Hyclone (Logan, UT, USA). Gentamicin, trifluoroacetic acid, α-cyano-4-hydroxy-cinnamic acid, Tris-HCl, NaCl, Tween-20 and Tween-80 were obtained from Sigma-Aldrich (Oakville, ON, Canada). Iodoacetamide, bis-acrylamide, ammonium persulfate, glycerol, immobilized pH gradient strips, Criterion Cassette (13.3 × 8.7 cm W x L), Tris/Glycine/SDS buffer, and BioSafeCoomassie Blue were purchased from Bio-Rad (Mississauga, ON, Canada). Trypsin, resazurin reduction (CellTiter-Blue®) and lactate dehydrogenase (LDH) cytotoxicity assay kits (CytoTox-96®) were from Promega Corporation (Madison, WI, USA), ATP assay kit (ViaLight™ Plus) was from Lonza Corporation (Rockland, ME, USA), and 5-bromo-2′-deoxyuridine (BrdU) cell proliferation ELISA (chemiluminescent) assay kit was obtained from Roche Diagnostics (Laval, QC, Canada). All materials were analyzed for endotoxin using the chromogenic Limulus amebocyte lysate assay (Lonza, Walkersville, MD, USA). All water used was deionized/demineralized (>16 MΩ resistivity). Water bath Branson 1510 sonicator was from Bransonic (Danbury, CT, USA), which provides an output of 70 watts and 42 kHz was used for all particle preparations and protein extraction purposes.

### Particle preparation

The urban dust EHC-93 was collected from baghouse air filters from the Environmental Health Centre in Ottawa, Ontario, Canada in 1993 and its preparation for toxicological studies has been described previously [57]. The water-soluble and water-insoluble fractions of EHC-93 were prepared as follows; a sample of EHC-93 was removed from a – 80 °C freezer and was warmed up to room temperature, 1 g of EHC-93 was placed in a clean 15 mL Falcon tube, re-suspended in 5 mL of sterile water and sonicated in a pre-chilled water bath for 20 min. The tube was then centrifuged (500×g, 10 min), and the aqueous supernatant was collected into another clean 15 mL Falcon tube. The pellet (insoluble) was resuspended in 5 mL of water, and this process was carried out three times to collect a total volume of 15 mL of aqueous supernatant. The pooled supernatant was further centrifuged (900×g, 3.5 h), the supernatant was collected and the remaining pellet was pooled with the insoluble materials. The aqueous supernatant was then filtered through a 0.2 μm nylon syringe-tip filter into a clean 50 mL Falcon tube. This filter was then washed with 5 mL of methanol and pooled with the aqueous suspension. The pooled aqueous suspension and the pooled pellet were then lyophylized and stored frozen at −80 °C. The final mass percentage recoveries of the particle fractions were 17% water-soluble (soluble) and 83% water-insoluble (insoluble).

To prepare the particles for dosing, the dried particulate materials from the total, insoluble and soluble fractions each were resuspended in particle preparation buffer (NaCl: 1.9 mg/mL or 32.5 mM; Tween-80: 25.0 μg/mL or 19.1 μM) in a Dounce glass-glass microhomogenizer. The final concentrations of the total, insoluble and soluble fractions were prepared according to their mass percentages (i.e., 10.0, 8.3 and 1.7 mg/mL, respectively). In this manner, the cytotoxicities of the insoluble and soluble fractions relative to the total can be directly assessed. The suspensions were sonicated on ice for 20 min and then dispersed as much as possible by 25 strokes of the homogenizer piston. Particle suspensions were then aliquotted into sterile, O-ring seal microcentrifuge tubes, heated to 56 °C in a water bath for 30 min, and were subsequently frozen at −80 °C until use. All materials were analyzed for endotoxin using the chromogenic Limulus amebocyte lysate assay (Lonza, Walkersville, MD, USA).

### Scanning electron microscopy (SEM)

The size and morphology of EHC-93 was characterized by SEM. Images were collected on a JSM-7500F FESEM (JEOL) instrument equipped with a Field Emission Gun (FEG) under the following parameters: beam acceleration voltage, 2 KV; working distance, between 7 and 9 mm; imaging mode, Lower Secondary Electron Image (LEI). Magnification and sizing bar are as indicated in the figure captions for each individual image. Samples were prepared by dropping a small amount of powder onto an aluminum stage painted with carbon paint (Electron Microscope Sciences, (EMS)). The paint was allowed to dry for 20 min, and the excess powder was then removed by blowing the surface with compressed, dry air.

### Energy dispersive X-ray spectroscopy (EDX)

EDX spectra were collected using a JSM-7500F FESEM (JEOL) instrument with the following parameters: beam acceleration voltage, 20 KV; acceleration current, 10 mA; working distance, between 8 and 9 mm. Since this instrument is attached to the SEM purchased from JSM, sample analysis was run concomitantly with SEM imaging, and thus, sample preparation for the collection of EDX spectra is identical to that for SEM. It should be noted that the carbon content in the sample cannot be determined because the stage is coated with carbon paint. The weight percent and atomic percent results were produced automatically from the instrument software analysis package.

### Inductively coupled plasma–mass spectrometry (ICP-MS)

Elemental analysis on EHC-93 total and its insoluble and soluble fraction were analyzed by ICP-MS in a previous report [58]. The results that are pertinent to this manuscript were summarized in Additional file 1: Table S1.

### Powder X-ray diffraction (pXRD)

The powder X-ray diffraction plot was obtained with a Rigaku Ultima IV instrument, equipped with a Cu tube. The powder sample was pressed by hand into a custom sample holder, such that a flat powder sample with a specified surface height would be presented to the X-ray beam. The pXRD plot was then collected in the 2 to 70 2theta degree range in continuous-scan mode, with a sample width of 0.02 degrees, and a scan speed of 0.25 deg./min. Percent distribution was measured following identification and integration of the peak areas for each crystal phase observed in the spectrum.

### Cell culture and particle exposure

The A549 cell line (American Type Culture Collection - CCL-185; human, epithelial, lung carcinoma) was subcultured in DMEM supplemented with 50 μg/mL gentamicin and 10% FBS. It should be noted that final FBS concentration the cells are exposed to is 5% after dosing with particles (particle preparations that were used to dose the cells were in serum-free media, then they were added to the 10% FBS culture media that contained the cells). The cells were maintained and subcultured in T-75 flasks in a humidified atmosphere containing 5% CO_2_ and 95% air at 37 °C. For exposure experiments, the cells were seeded at 1.5 × 10^6^ cells/T-25 flask (for proteomics) or 2.0 × 10^4^ cells/well in 96-well plate (for cytotoxicity assays), incubated for 24 h, resulting in approximately 75% confluence prior to dosing with particles. The final volume of culture medium was 5 mL (T-25), 15 mL (T-75) or 200 μL/well (96-well plate). Solutions of particles were prepared from frozen stocks, which were thawed to room temperature, sonicated on ice (20 min), then diluted in the culture medium to generate dosing concentrations that are equivalent to 0, 60, 140 and 200 μg/cm^2^ of the total (i.e., 0, 50, 116 and 166 μg/cm^2^ for the insoluble fraction and 0, 10, 24 and 34 μg/cm^2^ for the soluble fraction). The exposures were performed in this proportional manner so that the contributions of the insoluble and soluble components in EHC-93 total can be directly compared. However, the concentrations for both insoluble and soluble fractions were expressed in equivalent concentrations to the total in all the tables and figures in this study in order to assess the relative impacts of the two fractions. The cells were exposed to the particles by replacing the existing culture medium with the particle-containing medium, and the flasks/plates were returned to the incubator for a 24 h exposure to the particles. To harvest the exposed cells, the medium in each flask was removed and the cells were detached from the flasks using a plastic scraper. The cell suspension was collected in cell culture medium and centrifuged at 350 x g for 5 min, and the supernatant was discarded. The cell pellet was then washed twice with phosphate buffer saline (PBS). The final cell pellet was aspirated dry and stored at −80 °C until further use for proteomic analysis. This experiment was conducted in triplicate (*n* = 3) for all treatments.

### Integrated cytotoxicity assays

The integrated cytotoxicity bioassays which combined endpoints of cell viability (resazurin reduction assay), cellular membrane integrity (intracellular LDH release), and energy metabolism (ATP assay) were conducted in a single 96-well plate as described in a previous study [25]. The assays were carried out in the following sequence; after 24 h of exposure to particles, 100 μL of cell culture supernatant was transferred in a clear 96-well plate and clarified at 300 x g for 5 min (room temperature); 25 μL was used for LDH assay, 75 μL was frozen for other assays such as cytokine assays. Then, 50 μL of resazurin reduction reagent, prepared in culture medium (40% *v*/v), was added to the remaining 100 μL of culture medium and the cells were incubated (5% CO_2_, 37 °C) for 2 h. Aliquots (20 μL) were taken for measurement of resazurin reduction at 10 min and 120 min as described below. The cell culture supernatant was discarded by aspiration and the cells were lysed with 200 μL of lysis buffer (100 mM MgCl_2_ and 0.025% Triton X-100 in PBS) at room temperature, for 10 min. The lysate was recovered in clean plates and clarified by centrifugation as above; 25 μL of lysate was used for LDH measurement, 50 μL was used for ATP measurement, and 100 μL was frozen for additional analyses. The cell proliferation (BrdU incorporation) assay was performed in a separate 96-well plate. For all assays, supernatants and cell lysates were clarified by centrifugation to prevent interference of particles in the assays. All cytotoxicity assays were conducted in quadruplicate (*n* = 4) for all treatments.

In the resazurin reduction assay, viable cells reduce a non-fluorescent redox dye resazurin (dark blue in color) to a fluorescent reaction product resorufin (pink in color), and nonviable cells lose metabolic capacity to convert the indicator dye. Mitochondrial, cytosolic and microsomal enzymes have been implicated in the reduction of resazurin [16]. For measurement of resazurin reduction, 20 μL of supernatant aliquots at 10 and 120 min were transferred into clean plates containing 80 μL of serum-free medium per well, shaken at 350 rpm for 30 s on a circular plate shaker, and clarified by centrifugation at 300 x g for 5 min. Fluorescence of the diluted supernatants was measured by top reading at λ_Ex_ = 540 and λ_Em_ = 600 nm (Synergy 2, BioTek, Winooski, VT, USA). Resazurin reduction is calculated by fluorescence at 120 min minus fluorescence at 10 min.

The CytoTox 96® colorimetric assay quantitates the activity of cytosolic LDH released extracellularly during cell membrane damage (an indicator of cell death). The enzymatic activity released in the cell culture supernatants and recovered in the lysis buffer was measured with a coupled enzymatic reaction. LDH catalyzes the oxidation of lactate to pyruvate that is accompanied with the reduction of NAD^+^ to NADH, which in turn is consumed simultaneously in a diaphorase-catalysed reduction of tetrazolium salt, generating a soluble red formazan that can be detected by absorbance at 490 nm. For the assay of released LDH, 25 μL of the cell supernatants were combined with 25 μL of cell culture medium and 50 μL of LDH substrate from the assay kit. Absorbance at 490 nm (Synergy 2) was measured after 20 and 40 min of incubation in the dark. For the assay of cellular LDH, 25 μL aliquots of the cell lysates was combined with 25 μL of lysis buffer and 50 μL of substrate from the LDH assay kit. Absorbance at 490 nm was measured immediately and after 10 min of incubation in dark. The relative cellular LDH was calculated as a fraction of total LDH, that is LDH activity in cell lysate was divided by total LDH activity recovered in supernatant and cell lysate.

The ViaLight Plus is a bioluminescent assay for measurement of cellular ATP. Cell injury leading to mitochondrial perturbation results in a decrease of cellular ATP. In the presence of ATP and oxygen, the luciferase enzyme oxidises luciferin to oxyluciferin that accompany with photons emission. Chemiluminescence in the assay is proportional to the concentration of ATP in the cell lysate. The ATP working reagent was prepared 15 min prior to conducting the assay by mixing ATP monitoring reagent and the assay buffer provided in the kit, where 50 μL of the cell lysate was added to 100 μL of freshly prepared ATP reagent in a white-walled 96 well plate. Luminescence was measured (Synergy 2, Biotek) following 2 min incubation in the dark.

The BrdU Cell Proliferation ELISA is an enzyme immunoassay based on the incorporation of the thymidine analog BrdU during DNA synthesis in proliferating cells. Cells were grown in black-walled 96-well plates and exposed to particles for 24 h as described above. The BrdU labelling medium (10 μM BrdU) was added to each well, followed by a 4 h incubation (5% CO_2_, 37 °C). The medium was discarded and the plates were dried at 60 °C for 1 h, and stored at −40 °C until use. The cell monolayers were fixed with 200 μL of the fixation-denaturation reagent for 30 min and then incubated with anti-BrdU antibody for 2 h at room temperature. The wells were washed three times with 150 μL of PBS containing 0.01% Tween-80, and the substrate provided in the BrdU ELISA kit was added. The plates were covered with black tape and were shaken for 4 min. Chemiluminescence was measured (Synergy 2) with 1 s integrated readings per well.

### ELISA-based secretory cytokine assays

Levels of cytokines from the supernatant were measured by a Millipore MAP 8-plex human cytokine panel (EMD Millipore, Billerica, MA). The simultaneous quantification of cytokine levels was carried out according to Millipore recommended procedure, where a panel of 11 cytokines were assessed (GM-CSF, IL-1β, IL-1RA, IL-6, IL-8, IL-9, IL-10, IL-12p70, MCP-1, TNFα and VEGF), where the procedure was carried out in a Bio-Rad Bioplex 200 array reader instrument (Bio-Rad Laboratories, Mississauga, ON). Briefly, cell supernatants were thawed on ice and centrifuged at 956 x g for 5 min, at 4 °C. Next, 25 μl of samples were incubated with 25 μl of microbeads labeled with antibodies to the specific cytokines in a 96-well flat-bottom plate overnight at 4 °C. After the incubation the samples were washed twice using Bio-Rad Bioplex Pro II wash system, followed by incubation with the 25 μl of detection antibody cocktail for 1 h at room temperature (RT). The beads were then incubated with 25 μl of streptavidin-phycoerythrin for 30 min at RT, washed twice, and suspended in 150 μl of sheath fluid. The data were analyzed using the Bio-Rad Bio-Plex ManagerTM version 6.0 software, with 5PL curve fit and background fluorescence subtraction. The analysis was conducted in pooled samples of 3 wells per sample within each experiment, in quadruplicate experiments (*n* = 4). Cytokine levels in cell supernatants were determined from cytokine standard curves included on each plate. Only those cytokines that were detected at a concentration > 5 pg/ml in all data points would be used to filter out noises in the data. The final levels of cytokines for each treatment was adjusted to the viability of cells based on cellular LDH, BrdU incorporation and cellular ATP assays as previously described [8]. Data were expressed as normalized fold-change (FC) relative to the control (0 μg/cm^2^).

### Protein extraction and two-dimensional gel electrophoresis (2D–GE)

Total protein from the A549 cells (control & particle-exposed) was extracted and subjected to 2D–GE as previously described [62, 63]. Following electrophoresis, the gel was washed for 30 min in water, stained in BioSafeCoomassie Blue (Bio-Rad) overnight (16–20 h), destained twice in water (20 min), and then imaged with a standard scanner. To overcome the typical warping and distortion issues from gel to gel especially near the extremities of the pH range and the molecular weight, a common area across all experimental gels that clearly shows the protein spots was selected to assess the proteome differences among the treatments, where proteins in the window of pH 5.1–7.8 and 100–20 kDa were analyzed [62, 63]. A total of 543 well-resolved protein spots in this common area were compared across all experimental gels, and the identities of 333 of these protein spots were determined using MALDI-TOF-TOF-MS [62, 63]. The protein spots within the gels were matched and quantified with PDQuest™ Advance V8.0.1 (Bio-Rad), where spot volume was quantified using the available “Local regression model (LOESS)” algorithm in PDQuest. The reported spot volume for each protein was used to compare its level of expression across the treatments. In order to calculate the fold change for a protein spot from a treatment group, the treatment/control ratio (*n* = 3) was first determined. If the treatment/control ratio is between 0 and 1.0, a decreased expression (e.g., 0.5), then the fold-change is calculated by dividing “– 1.0” by the treatment/control ratio (e.g., − 1.0 / 0.5 = − 2.0). If the treatment/control ratio is >1.0, corresponding to increased expression (e.g., 1.5), then this serves as the fold-change by itself (https://www.qiagen.com/). Such fold-change values are used for bioinformatic analyses (e.g. IPA) as reported in Additional file 2: Table S2 and Figs. 4, 5, 6, 7. It should be noted that there is no value between “– 1.0 and 1.0” when the fold-changes are expressed in this manner. For hierarchical cluster analysis, however, fold-changes were calculated based on Log_2_(treatment/control) so that the data is continuous (i.e., there is no gap between – 1.0 and 1.0) for the appropriate analysis.

### Statistics

Hierarchical cluster analysis was conducted using GenePattern [37], and the resulting heatmap was generated with Java TreeView (https://sourceforge.net/projects/jtreeview/). Two-way analysis of variance (ANOVA) was performed on 2D–GE (*n* = 3), cytotoxicity assays (*n* = 4) and cytokine releases (n = 4) data with treatment and dose as factors, using R [35]. When the assumptions of equal variance or normality were not met, the data were rank transformed. Holm-Sidak was the *post-hoc* method used for all pairwise comparison procedures, which is a step-down procedure on a sorted set of null hypotheses. The reported *p*-values have been adjusted for the familywise error rate (FWER) which is the probability of making at least one type I error (incorrect rejection of a true null hypothesis) in the set or family of null hypotheses. A protein was considered as having a significant effect if the Holm-Sidak adjusted *p*-value was less than 0.05. If the *Treatment* x *Dose* interaction was significant for a protein spot, its change in expression for a given treatment and dose that was found significant by Holm-Sidak analysis was reported as it is, as presented in Additional file 2: Table S2. The same applied for those proteins that were found to have significant *Treatment* and *Dose* main effects. If a protein was found to have significant *Treatment* main effect, fold changes were estimated using least square mean [17, 43]. In the case where the *Dose* main effect was significant, the average FC estimate was reported for each significant dose group.

### Bioinformatics

It should be noted that multiple protein spots with the same protein ID may have a *p*-value <0.05, which suggests different isoforms of the same protein were significantly altered (Additional file 2: Table S2). When this was the case, selection for pathway analysis was based on the following order: best matching MW, largest spot volume, highest MOWSE score (molecular weight search) and then greatest FC. Furthermore, protein spots that were deemed as small peptides/fragments (based on MW and unique peptide sequences) of their native proteins were excluded from pathway analysis, unless functional data can be found for such peptides based on UniProt (www.uniprot.org) and PubMed (http://www.ncbi.nlm.nih.gov/pubmed) searches. It should be mentioned that a few cleaved protein products were included in pathway analysis in this study because they are known to serve functional purposes. For example, the precursor of HTRA2 is a 50 kDa mitochondrial membrane protein that became a mature serine protease of 36 kDa (SSP6208, see Additional file 2: Table S2) in the cytosol after 133 of its N-terminal amino acids has been proteolytically cleaved [50], where the mature peptide serves as an inhibitor of XIAP and IAPs [50]. Furthermore, an arbitrary ±1.10 FC cut-off was also applied on all significant proteins (adjusted *p*-value <0.05) when conducting pathway and network analyses. Protein interaction network and pathway analyses were conducted using Ingenuity Pathway Analysis (www.ingenuity.com).

## Results

### Physicochemical characterization of the EHC-93 particles

**Fig. 1:**
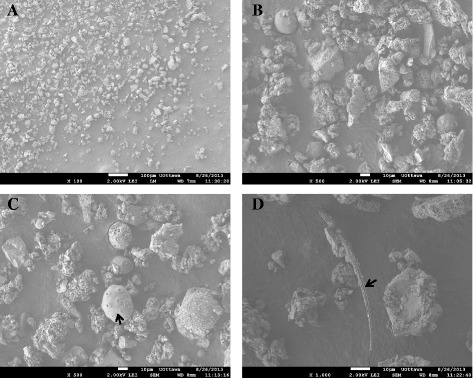
The particles in the EHC-93 sample observed by scanning electron microscopy at various magnifications to show the contents in the particulate matter. **a** is a 100X magnification image of the particles (scale bar is 100 μm). **b** and **c** are 500X magnification images of the particles (scale bars are 10 μm) that shows the majority of the particles have the appearance of mineral particles, where the arrow in **c** points to a spherical ordered particle (biological origin). **d** is a 1,000X magnification image (scale bar is μm), and it shows a thin rod (arrow) in the middle of the image

The electron micrographs in Fig. 1 show that the EHC-93 Ottawa urban dust is a complex mixture of particles with a broad range of size, shape, crystallinity, aggregation, porosity and surface structure. Majority of the materials appear to be crystalline particles with a wide range of sizes, where most particles possess flat non-porous plains and sharp edges. Some particles found in small quantities appeared as long thin rods (Fig. 1d), and as spherical non-porous, spherical porous and spherical ordered porous (biological origin) (Fig. 1c) materials. X-ray diffraction data in Table 1 showed that calcite (CaCO_3_), α-quartz (SiO_2_), gypsum (CaSO_4_) and dolomite (CaMg(CO_3_)_2_) were the major crystalline particles in EHC-93, which constituted of 41, 18, 13 and 13% of the crystalline particles, respectively. The EDX analysis revealed that Ca (31.9% by mass), Si (23.2% by mass) and S (11.4% by mass) are the three dominant elements in EHC-93 (Table 2). This EDX result is similar to that of the IPC-MS result from a previous study which also showed that Ca and Si are major elements in EHC-93 total (Additional file 1: Table S1) [58]. The combined results suggested that majority of the insoluble components in EHC-93 are calcite followed by α-quartz and gypsum.

**Table 1:** The percentage distribution of the major mineral crystals in the EHC-93 Ottawa urban dust detected by X-ray diffraction

Mineral crystal	% Distribution
Calcite (CaCO_3_)	41
α-quartz (SiO_2_)	18
Gypsum (CaSO_4_)	13
Dolomite (CaMg(CO_3_)_2_)	13
Albite (NaAlSi_3_O_8_)	10
Halite (NaCl)	5

**Table 2:** The percentage distribution of the major elements in the EHC-93 Ottawa urban dust detected by energy dispersive X-ray spectroscopy

Element	Weight %	Atomic %
Na	6.99	9.96
Mg	2.72	3.66
Al	6.46	7.84
Si	23.17	27.04
S	11.41	11.66
Cl	7.74	7.16
K	3.70	3.10
Ca	31.89	26.08
Fe	5.94	3.49

The results in Table 3 showed that EHC-93 total particles contain a very small amount of endotoxin (100.0 EU/kg material). Almost all of the endotoxin was found in the insoluble fraction of EHC-93 (91.6 ± 1.4 EU/kg equivalent mass to the total). Only a trace quantity of endotoxin can be found in the soluble fraction of EHC-93 (2.5 ± 1.0 EU/kg equivalent mass to the total).

**Table 3:** Endotoxin levels in EHC-93 total and its water-insoluble and soluble fractions

PM	Endotoxin (EU/kg material^a^)
Total^b^	100.0 ± 1.0
Insoluble	91.6 ± 1.4
Soluble	2.5 ± 1.0

^a^The quantity of endotoxin unit (EU) was expressed relative to EHC-93 total for direct comparison (i.e., 91.6 and 2.5 EU can be detected from 0.83 and 0.17 kg of materials from the insoluble and soluble fractions, respectively)

^b^Data has been published [8]

### Cytotoxic effects of EHC-93, and its insoluble and soluble components in A549 cells

**Fig. 2:**
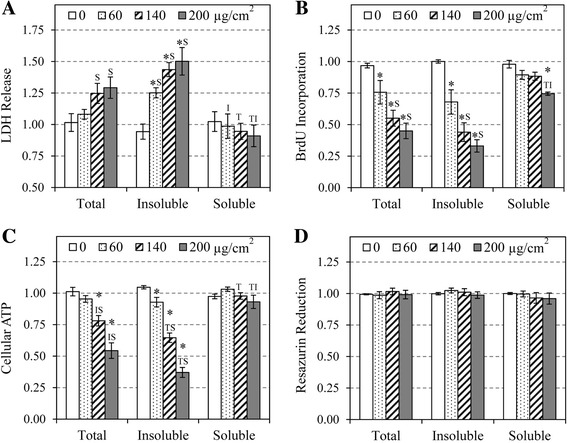
The cytotoxicities of EHC-93 total and its water-insoluble (insoluble) and water-soluble (soluble) fractions in A549 cells after 24 h of exposure were assessed by LDH release (**a**), BrdU incorporation (**b**), cellular ATP (**c**) and resazurin reduction(**d**) assays. Data are expressed as mean fold effect +/− standard error, relative to the control (0 μg/cm^2^), *n* = 4. Two-way ANOVA was used to determine significant effects of the particles, where Holm-Sidak was the post-hoc method used for all pairwise comparison procedures. * indicates significant difference compared to control. ^T^ indicates significant difference compared to EHC-93 total. ^I^ indicates significant difference compared to the insoluble fraction. ^**S**^ indicates significant difference compared to the soluble fraction

The cytotoxicity assays in Fig. 2 indicated that EHC-93 Ottawa urban air particles (total) had mild cytotoxic effect on A549 cells at low level of exposure (60 μg/cm^2^). However, EHC-93 total were cytotoxic to A549 cells at higher doses (140 and 200 μg/cm^2^), where they were capable of causing significant damage to the cell membrane based on LDH release assay (Fig. 2a), reducing cell proliferation based on BrdU incorporation assay (Fig. 2b) and decreasing metabolic energy content based on cellular ATP assay (Fig. 2c). Resazurin reduction assay did not detect any significant effect by EHC-93 total or its sub-fractions (Fig. 2d). It was observed that the trend of cytotoxicity of the insoluble fraction was remarkably similar to that of total PM, suggesting that the insoluble components drove most of the toxic effects of EHC-93 in A549 cells. Nevertheless, subtle cytotoxicity differences between the insoluble fraction and total PM can be observed in most assays, where the insoluble components appeared even more potent than EHC-93 total, and a significant difference between the two exposures was observed in the level of cellular ATP at the highest dose (two-way ANOVA: *Treatment x Dose* interaction at 200 μg/cm^2^, *p* < 0.05) (Fig. 2c). The soluble materials were relatively non-toxic to A549 cells, and their effects were significantly different than the total and insoluble components in most assays.

### Changes in the expression of proteins in A549 cells following exposures to EHC-93 and its insoluble and soluble fractions

Two-way ANOVA results in Additional file 2: Table S2 indicated that 206 protein spots were differentially altered significantly by the treatments (adjusted *p*-value <0.05), and 154 of these protein spots have been identified via MALDI-TOF-TOF-MS. The effects of particle treatments on most of these protein spots (i.e., 126 out of 154 identified proteins) were particle-specific (i.e., *Treatment* main effect, *Treatment* & *Dose* main effects, and *Treatment X Dose* interaction). It should be kept in mind that two-way ANOVA results are meant to identify significant differential changes in protein expression among the treatments, and these changes are not always significantly different from the control. For example, all of the *Treatment* main effects in Additional file 2: Table S2 were not due to significant difference from the control after particle exposures. Rather, most of the *Treatment* main effects were significant differences between the soluble fraction and the total and/or insoluble fraction based on Holm-Sidak multiple pair-wise comparison tests (adjusted *p*-values were not shown), and some were due to differences between the total and insoluble fraction. On the other hand, significant differences from the control as well as among the treatments can be identified by *Treatment* & *Dose* main effect and *Treatment X Dose* interaction as demonstrated in Additional file 2: Table S2. Holm-Sidak multiple pair-wise comparison tests showed that EHC-93 total and its insoluble fraction affected the expression of most protein spots similarly (e.g., same direction of expression), and that their effects were different from the soluble materials (e.g., opposite directions of expression) (Additional file 2: Table S2). Despite their similarity, differences between the total and insoluble fraction can be identified based on their FCs and adjusted *p*-values (not shown) following Holm-Sidak analysis. It should be noted that the two-way ANOVA results in Additional file 2: Table S2 revealed multiple significant protein spots with the same protein ID, an indication of different isoforms of the same protein and/or post-translational modification of the native protein. A large portion of the significant protein spots were small fragments of their native proteins (based on MW and unique peptide sequences).

**Fig. 3:**
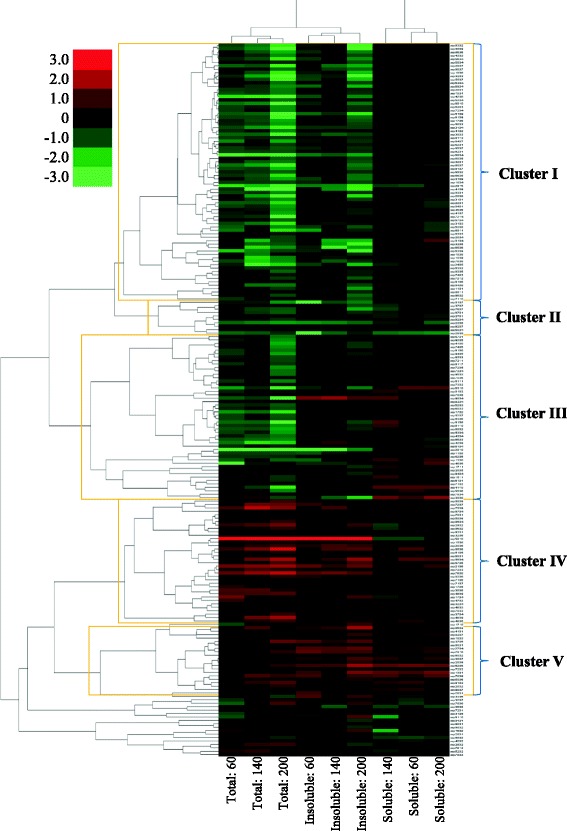
Unsupervised hierarchical cluster analysis of the protein spots that were significantly affected due to particle exposures (Two-way ANOVA: *p* < 0.05). The expression of each protein spot was calculated by Log_2_(Treatment/Control), *n* = 3. Red is coded for increased expression and green is coded for decreased expression. The number indicates the dose in μg/cm^2^. The involvment of the proteins in different clusters in various cellular functions is listed in Additional file 4: Table S3

Hierarchical cluster analysis was conducted to visually compare changes in the proteome of A549 cells following 24 h of exposure to EHC-93 and its insoluble and soluble fractions. The results based on all 543 protein spots examined by 2D–GE (Additional file 3: Figure S1) or only the significantly altered protein spots (Fig. 3) showed that the insoluble fraction and EHC-93 total formed a cluster that is separate from the soluble fraction. Such observations indicated that the total EHC-93 mixture and its insoluble components affected the proteome of A549 cells similarly, and that their effects differed from those of the soluble materials. It should be noted that the effects of the total PM and the insoluble were different enough that the two treatments formed two separate sub-clusters (Additional file 3: Figure S1 and Fig. 3). The significantly altered protein spots in Fig. 3 appeared to form five interesting clusters (I – V). Cluster I was dominated by those protein spots that were down regulated by the total and insoluble fraction; and these proteins were found to be involved in cellular movement, cell growth and proliferation, cell death and survival, molecular transport and small molecule biochemistry pathways (Additional file 4: Table S3). Cluster II was a small group of protein spots that were decreased in expression by all treatments, but the number of protein in this cluster was not large enough to conduct a reliable bioinformatics analysis. Cluster III displayed the protein spots that the total and insoluble fraction treatments generally decreased their expressions, while the soluble fraction generally increased their expressions; these proteins were involved predominantly in cellular movement, carbohydrate metabolism, cell growth and proliferation, cell death and survival, and cell morphology pathways (Additional file 4: Table S3). Cluster IV consisted of those protein spots that were strongly increased by most total and insoluble exposures but were weakly increased or decreased by the soluble treatments. The proteins in this cluster were found to be in the lipid metabolism, small molecule biochemistry, and cell growth and proliferation pathways. Cluster V showed the protein spots that were increased in expression by all treatments; these proteins are involved in cellular morphology, cellular function and maintenance, cellular assembly and organization, and cell death and survival.

### Effects of EHC-93 and its insoluble and soluble components on various pathways and networks in A549 cells

**Fig. 4:**
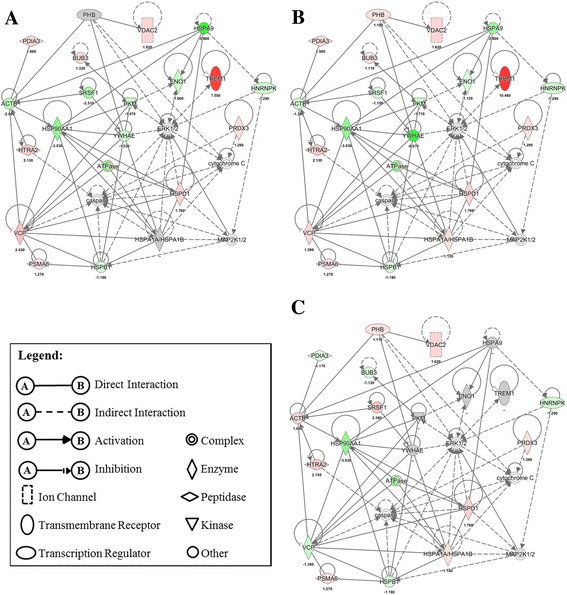
Protein profiles in the network of cell death and cell proliferation pathway in A549 cells following EHC-93 total (**a**) and its insoluble (**b**) and soluble (**c**) fractions treatments at 60 μg/cm^2^. Red indicates increased expression, green stands for decreased expression, grey implies non-significant change and white indicates the protein was not examined in this study. The color scale, representing fold-change, was set at a maximum and minimum of 8 (deepest red) and −6 (darkest green)

**Fig. 5:**
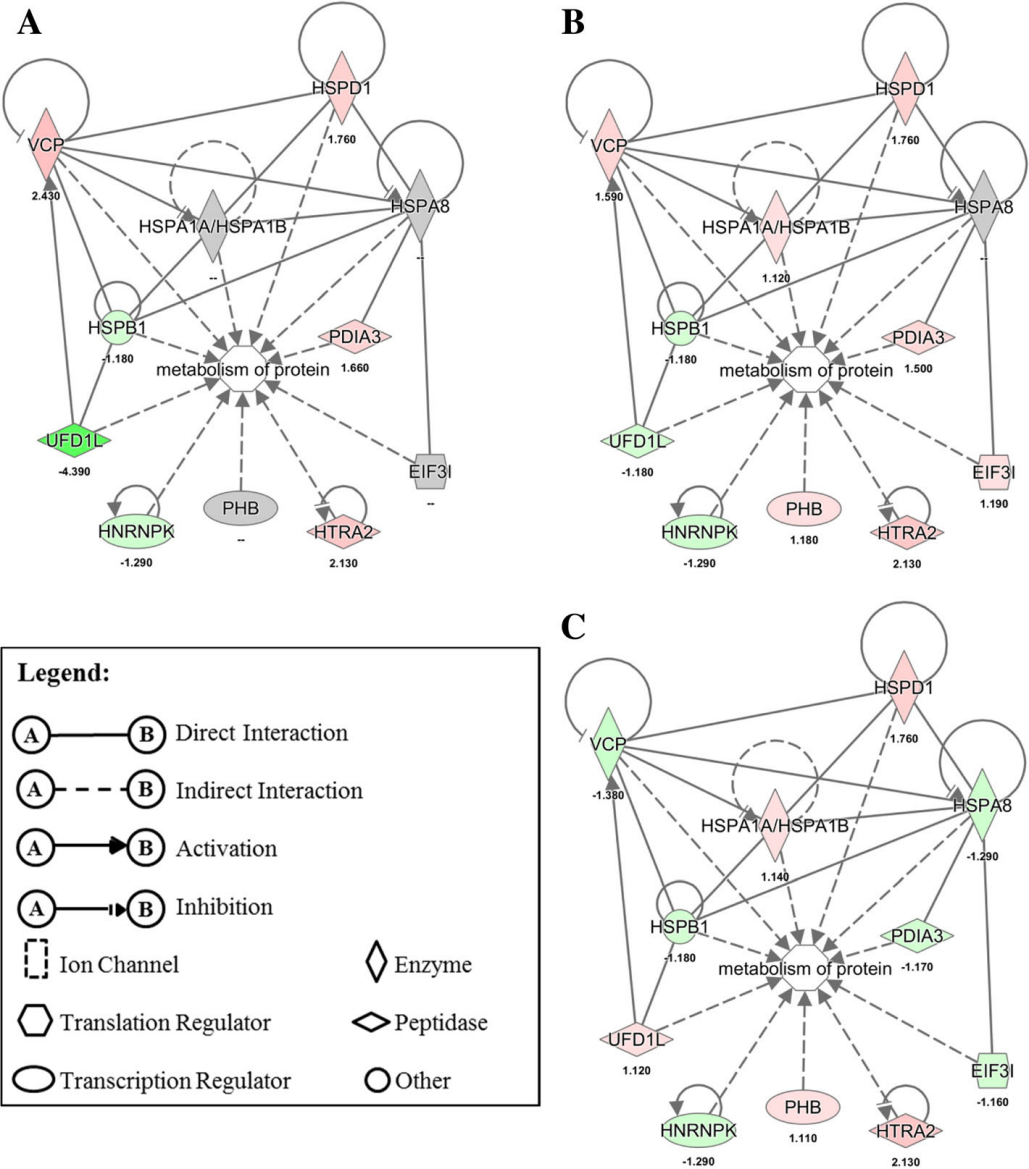
Protein profiles in the network of protein metabolism pathway in A549 cells following EHC-93 total (**a**) and its insoluble (**b**) and soluble (**c**) fractions treatments at 60 μg/cm^2^. Red indicates increased expression, green stands for decreased expression and grey implies non-significant change. The color scale, representing fold-change, was set at a maximum and minimum of 8 (deepest red) and −6 (darkest green)

**Fig. 6:**
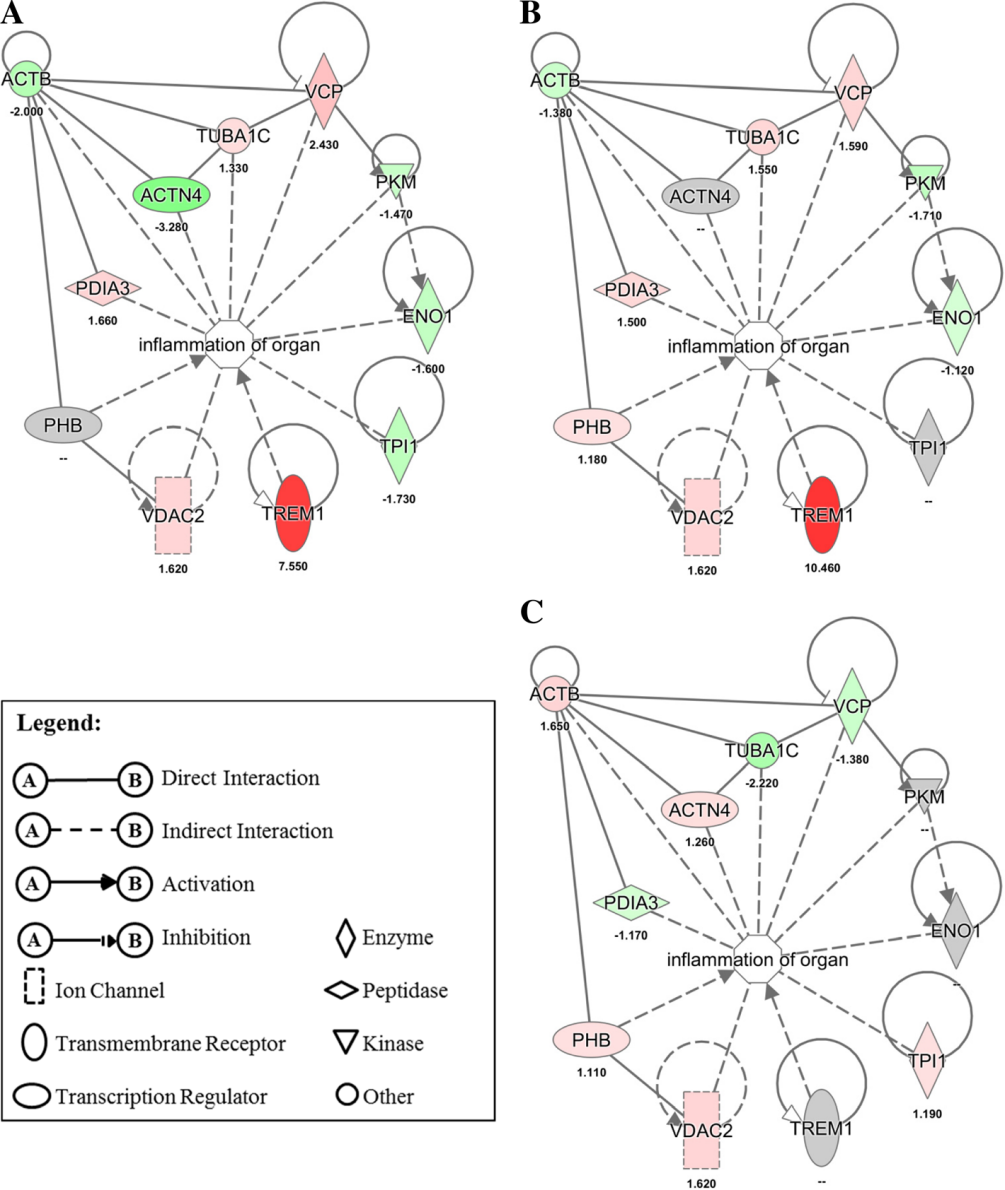
Protein profiles in the network of organ inflammation pathway in A549 cells following EHC-93 total (**a**) and its insoluble (**b**) and soluble (**c**) fractions treatments at 60 μg/cm^2^. Red indicates increased expression, green stands for decreased expression and grey implies non-significant. The color scale, representing fold-change, was set at a maximum and minimum of 8 (deepest red) and −6 (darkest green)

Ingenuity Pathway Analysis results in Table 4 revealed that EHC-93 and its insoluble and soluble fractions can affect the expression of the proteins involved in a number of biological functions including cell death, cell proliferation, cell differentiation, cellular movement, inflammatory response, protein metabolism and reactive oxygen species (ROS) metabolism. In these pathways, the patterns of protein expression in A549 cells influenced by the soluble fraction were noticeably different from the total and insoluble fraction, and the differences between the latter two are more subtle but distinguishable (Additional file 5: Figure S2). Generally, most of these proteins were altered by the total and insoluble fraction treatments in the same direction but varying in magnitude, whereas the soluble fraction exposure may cause no effect or opposite effects to that of the total and insoluble fraction treatments. For example, the networks of cell death and proliferation in Fig. 4 showed that the expressions of proteins such as YWHAE, SRSF1, PKM, HSPA9 and ENO1 were down-regulated in the total and insoluble fraction but were up-regulated or unaffected by the soluble fraction. The expression of proteins such as VCP, TREM1 and BUB3 were up-regulated in the total and insoluble fraction but down-regulated or unaffected in the soluble fraction. All these proteins were affected by the total and insoluble fraction in the same direction but varying magnitude. Similarly, the network of protein metabolism in Fig. 5 showed that the expression of PDIA3, HSPA8 and EIF3I were down-regulated due to the soluble fraction exposure, but these proteins were either up-regulated or unaffected by the total and insoluble fraction exposures to varying magnitude. The expression of UFD1L was up-regulated by the soluble fraction, but it was down-regulated by the total and insoluble fraction to different degrees. Of all the networks examined, the network of organ inflammation in Fig. 6 showed the most contrasting effect between the total or insoluble fraction against the soluble fraction, where 10/11 and 9/11 proteins in the network were distinctly altered, respectively. In this network, the total and insoluble fraction significantly increased the expression of PDIA3, TREM1, TUBA1C and VCP to various degrees in A549 cells, whereas exposure to the soluble fraction either did not affect or decreased the expression of these proteins. On the other hand, the expression of ACTB, ENO1 and PKM were significantly decreased in A549 cells to varying magnitude following exposure to the total and insoluble fraction, but these proteins were either unaffected or increased after exposing to the soluble fraction.

**Table 4:** Biological functions indicated by Ingenuity Pathway Analysis (IPA) that were likely impacted by the particles based on the proteins that were significantly affected

	Total		Insoluble		Soluble	
Biological Function	#	*p*-value	#	*p*-value	#	*p*-value
Cell Death and Survival	22	1.51 × 10^−05^	24	4.39 × 10^−09^	19	4.29 × 10^−05^
Cell Growth and Proliferation	21	2.42 × 10^−04^	22	6.91 × 10^−05^	18	5.85 × 10^−04^
Cellular Movement	17	1.83 × 10^−05^	18	3.89 × 10^−06^	16	6.56 × 10^−06^
Acute Inflammation	10	1.45 × 10^−03^	9	5.30 × 10^−03^	8	6.33 × 10^−03^
Chronic Inflammation	10	1.62 × 10^−04^	8	3.39 × 10^−03^	9	2.15 × 10^−04^
Cytoplasm Organization	9	2.03 × 10^−02^	10	6.95 × 10^−03^	9	6.90 × 10^−03^
Protein Metabolism	7	1.07 × 10^−02^	10	1.24 × 10^−04^	11	3.80 × 10^−06^
ROS Metabolism	6	1.40 × 10^−03^	8	2.74 × 10^−05^	5	3.83 × 10^−03^
Allergic Response	7	5.18 × 10^−05^	6	4.31 × 10^−04^	6	1.71 × 10^−04^
Nucleic Acid Metabolism	6	1.62 × 10^−03^	7	8.05 × 10^−05^	5	1.95 × 10^−03^
Mitochondrial Transmembrane Potential	5	1.22 × 10^−04^	6	8.52 × 10^−06^	5	5.47 × 10^−05^

It should be noted that about half of the proteins used in pathway analysis derived from *Treatment* main effect, where the effect of the soluble fraction on these protein spots were typically opposite that of the total and insoluble fraction. The directions of protein expressions of several selected functions were demonstrated as heatmaps in Additional file 5: Figure S2. The # indicate the number of proteins that were significantly affected by each treatment (EHC-93 total, insoluble and soluble at 60 μg/cm^2^), and the *p*-value indicate the significance of the biological function based on IPA’s calculations. Only the significant functions that were influenced by more than 5 proteins in any particle treatment group are presented

### Secretion levels of IL-8, MCP-1 and VEGF from A549 cells due to exposures to EHC-93 and its insoluble and soluble fractions

**Fig. 7:**
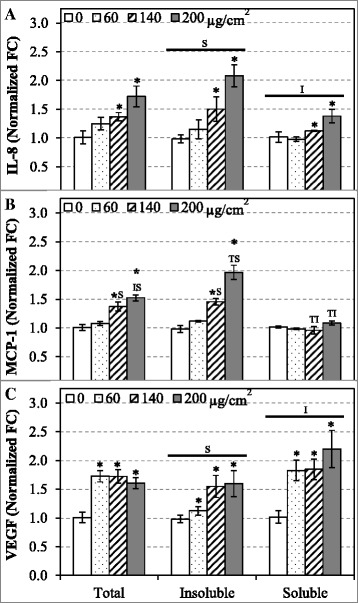
Comparing the secretion of cytokines such as IL-8 (**a**), MCP-1 (**b**) and VEGF (**c**) by A549 cells after 24 h exposure to EHC-93 total and its insoluble and soluble components. Data are expressed as normalized fold-change (FC) ± standard error, relative to the control (0 μg/cm^2^), n = 4. Two-way ANOVA was used to determine significant effects of the particles, where Holm-Sidak was the post-hoc method used for all pairwise comparison procedures. * indicates significant difference compared to control. ^T^ indicates significant compared to EHC-93 total. ^I^ indicates significant difference compared to the insoluble fraction. ^**S**^ indicates significant difference compared to the soluble fraction. The bar on top of a treatment group indicates significant *Treatment* main effect

From a panel of 11 cytokines assessed, only 3 cytokines (IL-8, MCP-1 and VEGF) were found to secrete at a reliable detection level (> 5 pg/ml) and were significantly altered due to particle exposures (Fig. 7). It was found that EHC-93 total and its insoluble fraction displayed a similar trend in stimulating the secretion of IL-8, MCP-1 and VEGF from A549 cells that is different from the soluble fraction. The insoluble fraction is significantly more potent than the soluble fraction in stimulating the releases of the pro-inflammatory cytokines interleukin-8 (IL-8) and monocyte chemoattractant protein-1 (MCP-1) from A549 cells (Fig. 7a and b). The insoluble fraction appeared more potent than the total in inducing the secretion of IL-8 and MCP-1 from A549 cells, and it reached statistical significant for MCP-1 at the highest dose (Fig. 7b). Contrarily, the soluble fraction is significantly more potent than the insoluble fraction in causing the release of vascular endothelial growth factor (VEGF) from A549 cells (Fig. 7c). The total and insoluble fraction can stimulate significant release of VEGF from A549 cells, but their potencies were not significantly different.

## Discussion

Understanding the mechanisms of particle toxicity of urban air particulate matter (PM) is a challenge, because airborne PM is a complex mixture of particles with a wide range of physicochemical properties. In an attempt to assess the in vitro toxicity of urban air particles, we previously fractionated EHC-93 (Ottawa urban air particles) into water-insoluble and soluble fractions, and used A549 cells to examine the PM exposure-related effects on a few selected genes and secretory proteins in the endothelin system [12]. The current study focuses on integration of multiple cytotoxicity assay and untargeted proteomic analysis results to gain insight into toxicity mechanisms underlying total and fractionated PM exposure-related changes in A549 cells.

This is the first time that multiple cytotoxicity assays were used to investigate the cytotoxic effects of EHC-93 and its insoluble and soluble fraction in the same in vitro study. The results from LDH release, cellular ATP and BrdU incorporation assays indicated that EHC-93 total and its insoluble fraction were both similarly cytotoxic to A549 cells, while the soluble fraction caused very little toxic effect to the cells (Fig. 2). It could be argued that the cells were actually dosed with low concentrations of soluble materials (0, 10, 24 and 34 μg/cm^2^), which were 17% mass equivalent to the dosing concentrations of the total (0, 60, 140 and 200 μg/cm^2^), and thus such low quantities of the soluble materials were not high enough to exhibit a significant effect. However, this is the actual proportion of the soluble materials present in EHC-93 total, and the present study focused on comparing the cytotoxic effects of the insoluble and soluble fractions in the mass proportion as they were found in the environment. Similarly, A549 cells were dosed with 0, 50, 116 and 166 μg/cm^2^ insoluble materials, which are 83% mass equivalent to EHC-93 total concentrations. Nevertheless, the insoluble fraction appeared even more toxic than the total in every equivalent dose of every cytotoxicity assay, and reached statistical significance at the highest dose in ATP assay (two-way ANOVA: *Treatment x Dose* interaction at 200 μg/cm^2^, *p* < 0.05) (Fig. 2c). Evidently, the toxic potency of EHC-93 total is not equal to the sum of its insoluble and soluble fractions, suggesting that there were interactions between the insoluble and soluble materials. It is possible that the soluble materials coated the surface of the insoluble components and reduced some of their cytotoxic effects. Such inhibitory coating effect has been previously reported in nano-silica particles [48]. Alternatively, biological effects caused by the soluble materials in A549 cells may be antagonistic to some of those effects elicited by the insoluble materials.

The effects of EHC-93 and its insoluble and soluble fractions on the proteome of A549 cells were examined via 2D–GE and mass spectrometry as previously described [62, 63]. Two-way ANOVA and Holm-Sidak (post-hoc) were the statistical methods used to determine significant effects of particles across treatments. The results in Additional file 2: Table S2 indicated that there was a significant *Treatment* main effect for BUB3 (SSP9301), where the total and insoluble components of EHC-93 affected the expression of BUB3 similarly but their effects were different from the soluble materials. On average, the expression of BUB3 in A549 cells due to EHC-93 total, insoluble and soluble materials exposures were listed as 1.32, 1.11 and −1.12, respectively. It is important to understand that such FCs were relative to the control. More importantly, it must be recognized that the net difference between the effects of the total and soluble fraction was 44% (from 1.32 to −1.12) and the net difference between the effects of the insoluble and soluble fractions was 23% (from 1.11 to −1.12). Thus, such magnitudes of changes between treatments should not be overlooked, particularly when the adjusted *p*-value is very small (adjusted *p* = 0.003), and especially the goal of this study is to identify the differential responses of A549 cells to EHC-93 particles and its insoluble and soluble components. When conducting pathway and network analyses, a ± 1.10 FC cut-off on all significant proteins (adjusted *p*-value <0.05) should be sufficient to remove nuanced expressions that may not contribute to any biological impact.

Two-way ANOVA results in Additional file 2: Table S2 identified 206 protein spots were significantly altered by the treatments (adjusted *p*-value <0.05), and 154 of these protein spots have been identified via MALDI-TOF-TOF-MS, which can potentially be used for pathway analysis. Unsupervised hierarchical cluster analysis based on all the significantly altered protein spots showed that the total and insoluble fraction treatments clustered together (Fig. 3), suggesting that these two treatments had similar effects on the proteome of A549 cells. Such result holds true regardless if cluster analysis was conducted based both non-significantly and significantly altered protein spots (Additional file 3: Figure S1). In addition, the total and insoluble fraction treatments formed separate sub-clusters, implying that their effects on the proteome of A549 cells were distinguishable (Fig. 3 and Additional file 3: Figure S1). These findings were similar to those in an earlier study in our laboratory that demonstrated that the expression of a selected set of genes (e.g., *MMP2*, *ECE1* and *EDN1*) and secretory proteins (e.g., IL-8 and VEGF) in A549 cells were similarly affected by the total and insoluble components of EHC-93, and that their effects differed from those of the soluble materials [12]. Results from two-way ANOVA and Holm-Sidak multiple comparisons analyses in Additional file 2: Table S2 were in line with the above observations. The total and insoluble fraction PM exposures were observed to alter the proteome of A549 cells more than the soluble fraction, and these changes were not additive. In brief, all proteomic and cytotoxicity assay results in this study unanimously pointed out that the toxic effects of EHC-93 in A549 cells were mainly driven by its insoluble components.

Unsupervised hierarchical cluster analysis revealed that all the doses of the same treatment clustered together, and the highest dose of the total and insoluble fraction exposures induced the greatest change to most protein spots (Fig. 3). These findings suggested that the effects of the total and insoluble fraction would eventually converge to the same outcome as the dose increases. Interestingly, majority of these significantly altered protein spots were not full length native proteins or isoforms based on their molecular weights and unique peptide sequences (Additional file 2: Table S2). These peptides (e.g., SSP2010, 8302, 3104, 4108, 305 and 8109) were possibly cleavage or degradation products of their native proteins, and they may be derived from dying or dead cells that have undergone or have committed to apoptosis or necrosis. This is plausible because functional annotation for the proteins in different clusters in Fig. 3 revealed that cell death and survival and cell growth and proliferation were the two dominant cellular functions affected by particle exposures (Additional file 4: Table S3). In addition, the LDH release, cellular ATP and BrdU incorporation assays indicated that A549 cells were adversely impacted at the higher doses (Fig. 2). Altogether, these results suggested that the A549 cells exposed to the higher doses of PM (140 and 200 μg/cm^2^) had undergone terminal stage of particle toxicity (i.e., dead or dying cells), while the lowest dose (60 μg/cm^2^) revealing an early stage of particle toxicity (i.e., live cells). It should be noted that the chosen exposure doses for most in vitro toxicology studies, including the current study, are well beyond the actual environmental levels in order to obtain measurable responses. Therefore, there is more value to examine the effect of the particles on A549 cells at the low toxicity dose to capture the early signs of particle toxicity.

Pathway analysis revealed that the significantly altered proteins in A549 cells following exposure to the particles at 60 μg/cm^2^ dose were involved in pathways such as ROS metabolism, inflammatory response, cytoplasm organization, cellular movement, cell growth and proliferation, and cell death and survival (Table 4). These pathways were likely the mechanisms employed by A549 cells to handle the effects of the exposed particles at a low toxicity level. It should be noted that about half of the significantly altered proteins in these pathways were derived from *Treatment* main effect, where their expressions were not necessarily different from the control significantly. Rather, their expression were mostly opposite in direction between the soluble fraction and the total or insoluble fraction (Additional file 5: Figure S2). It should be clarified that the proteomic results based on 2D–GE data did not have sufficient power to confidently determine if any of the pathways in Table 4 was actually activated or inactivated. Rather, we relied on the cytokine release (Fig. 7) and cytotoxicity assays (Fig. 2) data to determine the phenotypic effects of the particles.

It is interesting to note that a large number of significantly altered proteins were involved in the cell death and survival pathways (Additional file 5: Figure S2), suggesting that this dose (60 μg/cm^2^) was sufficient to cause such effects. This is consistent with the cytotoxicity assay results (Fig 2a–c). Examining the pattern of proteins expressed in the networks of cell death and cell proliferation pathways in Fig. 4 may provide insights to the molecular mechanisms that dictate the contrasting effects of the insoluble and soluble fractions. In this network, the extracellular signal-regulated kinase 1/2 (ERK1/2) was found in one of the main nodes, where this protein is known to modulate a broad biological functions in cells, including cell death and cell proliferation [38]. It was noticeable that the soluble fraction treatment did not significantly alter the expression of a number of proteins up- and down-stream of ERK1/2. For example, only the total and insoluble treatments significantly decreased the expression of 14–3-3 protein epsilon (YWHAE), pyruvate kinase (PKM) and enolase-1 (ENO1) and significantly increased the expression of triggering receptor expressed on myloid cells 1 (TREM1). Down regulation of ENO1 and PKM may explain the decreased ATP levels in A549 cells follow the total and insoluble fraction exposures in Fig. 2c (significant only in the insoluble fraction treatment at dose 60 μg/cm^2^) as these two proteins are known to serve distinct enzymatic functions in the last two steps of glycolysis [59]. Interestingly, these proteins are known to serve several other biological functions such as cell death, cell proliferation and stress response, and their expressions can be modulated in response to various stimuli [64, 68]. ENO1 has been reported to regulate the kinase activity of ERK1/2 in A549 cells [68], and ERK1/2 can modulate the nuclear translocation of PKM in U251 human glioblastoma cells that is necessary for PKM’s auto-regulation of expression [64]. Knockdown of PKM expression (via siRNA) has been shown to decrease the production of ATP and induce apoptosis and autophagy in A549 cells [13, 49], which is consistent with the LDH release and BrdU incorporation trends (Fig. 2a and b). These findings were similar to the proteomic results observed for the total and insoluble fraction exposures and were also consistent with the cytotoxicity data (Fig. 2c) in this study. Furthermore, PKM has been shown to phosphorylate the mitotic checkpoint protein BUB3, which is essential for the BUB3-BUB1 complex to be recruited to the kinetochore spindle during mitosis [22]. The expression of BUB3 in A549 cells was increased by the total and insoluble fraction treatments, but its expression was decreased by the soluble fraction (Fig. 4). It is evident that the cytotoxicity assay and proteomic results were mutually complementary in explaining the toxic effects of the particles, extensive investigations would be required to better understand the PM-driven mechanisms of particle toxicity in the cell death and cell proliferation pathways.

Of all the networks examined, the network related to inflammatory process in Fig. 6 showed the most contrasting effect between the total or insoluble fraction against the soluble fraction, where 10/11 and 9/11 proteins in the network were distinctly altered, respectively. It should be cautioned that the relationships of the proteins involved in this network are not straight forward to interpret because inflammation is a process that involves multiple cell types such as epithelial cells, neutrophils and macrophages. Interestingly, inflammatory stimuli are known to increase the expression of triggering receptor expressed on myeloid cells 1 (TREM1) on neutrophils, monocytes and macrophages, where this receptor is known to amplify the secretion of pro-inflammatory mediators such as IL-1β, IL-6, IL-8, MCP-1 and TNFα from these cells [6, 40]. Furthermore, TREM1 has been reported to be expressed in lung cancer epithelial cells. For instance, A549 cells exposed to silica nano-particles have been associated TREM1 signaling [31]. Increased expression of this receptor in the cells exposed to the total and insoluble fraction is a possible mechanism for the release of IL-8 and MCP-1 in A549 cells (Fig. 7). In addition, a recent mouse lung injury model demonstrated that increased expressions of protein disulfide-isomerase associated 3 (PDIA3) and ENO1 were important for alveolar epithelial type II (AT-II) cells to repair bleomycin-induced injury in the lung of mice [29]. Over-expression of PDIA3 in murine embryonic fibroblast cells is known to exacerbate apoptosis via Bak signaling [67]. Mutze et al. [29] and Zhao et al. [67] hinted that increased level of PDIA3 in injured cells may determine whether the cells would commit to injury repair or apoptosis. As increased level of PDIA3 (Fig. 6) in A549 cells in the present study coincided with increased LDH release and decreased BrdU incorporation and cellular ATP levels (Fig. 2) after exposure to the total and insoluble fraction at the 60 μg/cm^2^ dose, the results point perhaps to an inflammatory process directed-apoptosis. EHC-93 is known to contain inflammatogenic constituents such as silica (Table 1) and endotoxins (Table 3). Moreover, previous studies have shown that EHC-93 was capable of stimulating the release of pro-inflammatory cytokines such as IL-8 [8, 12, 14, 39] and MCP-1 [8] from bronchial or lung epithelial cells. Sakamoto et al. [39] demonstrated that the secretion of IL-8 from human epithelial bronchial cells by EHC-93 total was induced by the influx of calcium from the extracellular media, where the signalling was suspected to be mediated by a membrane receptor and/or ion channel [39]. Whether EHC-93 total or its insoluble components induced secretion of IL-8 and MCP-1 from A549 epithelial cells were mediated by calcium influx, and the involvement of a membrane receptor/ion channel, will be explored in future studies.

It should be noted that correlating the in vitro results in this study to those in vivo results from previous studies is not straight forward. When EHC-93 total particles were inhaled by rats, the inhalation did not result in lung injury but it elicited inflammatory responses and cardiovascular effects [7, 51, 56, 58]. These results suggested that the efficient clearance mechanism in the respiratory tract of healthy animals and humans would make the inhaled particles only mildly toxic. Intriguingly, the data based on in vitro and intratracheal instillation studies suggested that the particles can be toxic if they were deposited and retained in the lungs. For example, the in vitro results in the present study demonstrated that A549 human type II lung epithelial cells were sensitive to the cytotoxic effects of EHC-93 and its water-insoluble components upon exposure, where the particles are potent in stimulating proteins involved in inflammatory responses and cell death, while decreasing cellular ATP and cell proliferation. On the other hand, direct injection of either EHC-93, its soluble or insoluble fractions into the lungs of rats via intratracheal instillation triggered inflammation based on the number of cells and protein levels in lavaged lung fluid [2]. However, exposure to EHC-93 and its soluble, but not insoluble, fraction caused mild lung injury in rats based on the observed necrosis to type I alveolar cells and the subsequent ^3^H–thymidine uptake by type II alveolar cells [2], which would proliferate and differentiate to replace type I cells. It is not clear why the instilled insoluble fraction did not induce lung injury even when it contains varying amount of insoluble minerals (Table 1), endotoxins (Table 3), PAHs and metals [57]. It is possible that the insoluble particles were cleared from the lung. As for the instilled soluble materials, they can be readily absorbed by the cardiovascular system and affect various cell types, where the observed lung injury was attributed to the presence of soluble zinc and copper [1, 34]. In addition, the immunoassay results in this study showed that the soluble materials had greater potency than the insoluble materials in stimulating the secretion of a potent vasculogenic/angiogenic signaling protein vascular endothelial growth factor (VEGF) from A549 cells (Fig. 7c), which are type II alveolar cells. In this point of view, the effects of the soluble materials could be mediated by type II alveolar cells via paracrine signalling.

In summary, most of the results in this study and previous in vitro study [12] consistently showed that the cytotoxic effects of EHC-93(total) and its insoluble fraction in A549 cells were similar to each other and their effects were differed from the soluble fraction. These results indicated that the insoluble materials in EHC-93 are the drivers of toxic potency in A549 human lung epithelial cells. The culminated physicochemical characterizations from the previous [57, 58] and present studies have built a repertoire of identified insoluble components in EHC-93 including metals (e.g., iron, lead, magnesium and zinc), minerals (e.g., calcite, silica and gypsum), carbonaceous materials (e.g., phenanthrene, pyrene, fluoranthene and benzo[b]fluoranthene) and endotoxins at defined quantities or relative quantities. This repertoire would allow present and future studies to better assess the contribution of toxic potency and assess the pathways of effects of one or a combination of particles. The majority of the insoluble components are mineral crystal particles such as calcite (CaCO_3_), α-quartz (SiO_2_), gypsum (CaSO_4_) and dolomite (CaMg(CO_3_)_2_) (Table 1). As these insoluble mineral particles can contribute to the total cytotoxic effects of EHC-93, other minor insoluble components such as endotoxins (Table 3), metals (e.g., Fe, Al, Pb, Mg, Sn and Ti) (Additional file 1: Table S1) and PAHs [57] may also add significant cytotoxicity to A549 cells. The insoluble materials in EHC-93 affected markers of inflammatory responses (Figs. 6 & 7) as well as cell death and proliferation in A549 cells (Figs. 2 & 4 and Table 4). Importantly, the proteomic results in this study provided molecular details associated with the toxicity of EHC-93 and its insoluble fraction.

## Conclusions

To our knowledge, this is the first study that used in tandem multiple cytotoxicity assays and proteomic analyses to assess the phenotypic outcomes and molecular mechanisms of particle toxicity of an urban air PM (i.e., EHC-93) and its insoluble and soluble fractions on human lung epithelial cells (A549). Both cytotoxicity assays and proteomic results consistently indicated that the insoluble materials explained most of the toxic effects of the total PM. Furthermore, the toxic potency of EHC-93 total is not equal to the sum of its insoluble and soluble fractions, implying inter-component interactions between insoluble and soluble materials that may be reflected through synergistic or antagonistic in vitro responses. Finally, this study demonstrated that in vitro toxicoproteomics is a valuable tool in delineating the toxicity mechanisms of environmental air particles.

## Additional files

Additional file 1: Table S1. Elemental content of EHC-93 and its water-insoluble and soluble fractions were examined by IPC-MS [58]. Foot Note: It should be noted that the mass of each element presented did not take into account that the insoluble and soluble fractions corresponded to 83 and 17 mass % of the total. (DOCX 15 kb)

Additional file 2: Table S2. Two-way ANOVA results for the A549 protein spots that changed significantly due to particle exposures (*n* = 3). The SSP number corresponds to the identifier number that PDQuest used to identify the spot based on its coordinate in the gel. The number below *Treatment* main effect (Trt), *Dose* main effect (Dose) or interaction between *Treatment and Dose* (T x D) corresponds to the *p*-value, where the bolded number emphasized *p*-value < 0.05. Only the protein spots identified by MALDI-TOF-TOF-MS/MS are provided here ([61]; [62]). The proteins indicated in red (likely degradation product of the native protein) and fold-change indicated in blue (cut-off at ±1.10) were excluded from pathway analysis. Orange colored spots were used for pathway analysis in the 60 μg/cm^2^ dose. The yellow highlight shows multiple protein spots with the same protein ID. See the Materials and Methods section for more information on the protein spot selection criteria for pathway analysis. Foot Note: ^**§**^ Spot volume intensity normalized to the control (n = 3). ^**†**^Significant change in protein expression identified by multiple comparison based on Holm-Sidak method (see Materials and Methods), which was used for pathway analysis, and the blank entries imply non-significant changes as compared to the control (i.e., fold-change = 1.0). Those protein spots with *p*-value <0.05 (based on Two-way ANOVA) but did not pass Holm-Sidak test were excluded. (DOCX 1799 kb)

Additional file 3: Figure S1. Unsupervised hierarchical cluster analysis demonstrating the effect of all the tested particles on the proteome of A549 cells. The expressions of all the well-defined protein spots in the 2D gels were examined. The expression of each protein spot was calculated by Log_2_(Treatment/Control), *n* = 3. Red is coded for increased expression and green is coded for decreased expression. The number indicates the dose in μg/cm^2^. (DOCX 196 kb)

Additional file 4: Table S3. Top cellular functions in which the proteins in various clusters (in Fig. 3) were involved based on IPA. Only those functions that were significantly (*p* < 0.05) influenced by more than 5 proteins were presented. (DOCX 12 kb)

Additional file 5: Figure S2. Changes in the expression of proteins in various pathways in A549 cells that were exposed to EHC-93 total and its insoluble and soluble fractions (at 60 μg/cm^2^) examined by hierarchical cluster analysis. These selected pathways were based on the top biological functions identified by Ingenuity Pathway Analysis (in Table 4). The color scales that show fold-changes, Log_2_(Treatment/Control), were set between −3 to 3 in panels A – C and −2 to 2 in panels D – F. (DOCX 259 kb)

## Abbreviations

2D–GE: Two-dimensional gel electrophoresis
ANOVA: Analysis of variance
BrdU: 5-bromo-2′-deoxyuridine
DMEM: Dulbecco’s modified Eagle’s medium
EDX: Energy dispersive X-Ray spectroscopy
EHC-93: Ottawa urban air particles collected from the Environmental Health Centre in 1993
EU: Enzymatic unit
FBS: Fetal bovine serum
FC: Fold-change
FWER: Familywise error rate
ICP-MS: Inductively coupled plasma–mass spectrometry
LDH: Lactate dehydrogenase
MALDI-TOF-MS: Matrix-assisted laser desorption ionization-time of flight-mass spectrometry
MOWSE: Molecular weight search
MS: Mass spectrometry
MW: Molecular weight
PBS: Phosphate buffer saline
pI: Isoelectric point
PM: Particulate matter
pXRD: Powder X-ray diffraction
ROS: Reactive oxygen species
RT: Room temperature
SEM: Scanning electron microscopy
