# Supplementary material for: In vitro toxicoproteomic analysis of A549 human lung epithelial cells exposed to urban air particulate matter and its water-soluble and insoluble fractions
Source: Part Fibre Toxicol. 2017 Oct 2;14:39. doi: 10.1186/s12989-017-0220-6 (PMC5625787; doi:10.1186/s12989-017-0220-6)
Supplement: Supplementary file 1 — Elemental content of EHC-93 and its water-insoluble and soluble fractions were examined by IPC-MS [58]. Foot Note: It should be noted that the mass of each element presented did not take into account that the insoluble and soluble fractions corresponded to 83 and 17 mass % of the total. (DOCX 15 kb) [file 12989_2017_220_MOESM1_ESM.docx]

**Table S1**. Elemental content of EHC-93 and its water-insoluble and soluble fractions were examined by IPC-MS (Vincent et al., 2001).

|  | **Total** | | **Insoluble** | | **Soluble** | |
| --- | --- | --- | --- | --- | --- | --- |
| **Element** | **(μg/g)** | **Mass %** | **(μg/g)** | **Mass %** | **(μg/g)** | **Mass %** |
| Aluminum (Al) | 23,700 | 7.3 | 25,400 | 8.1 | 650 | 0.6 |
| Barium (Ba) | 411 | 0.1 | 470 | 0.1 | 123 | 0.1 |
| Boron (B) | 61 | 0.0 | 48 | 0.0 | 20 | 0.0 |
| Cadmium (Cd) | 23 | 0.0 | 8 | 0.0 | 16 | 0.0 |
| Calcium (Ca) | 122,000 | 37.7 | 114,000 | 36.3 | 65,000 | 64.6 |
| Chromium (Cr) | 70 | 0.0 | 71 | 0.0 | 0 | 0.0 |
| Cobalt (Co) | 11 | 0.0 | 9 | 0.0 | 2 | 0.0 |
| Copper (Cu) | 763 | 0.2 | 742 | 0.2 | 500 | 0.5 |
| Iron Fe) | 20,200 | 6.2 | 22,500 | 7.2 | 350 | 0.3 |
| Lead (Pb) | 6,780 | 2.1 | 7,210 | 2.3 | 1,500 | 1.5 |
| Magnesium (Mg) | 15,200 | 4.7 | 15,800 | 5.0 | 3,000 | 3.0 |
| Manganese (Mn) | 594 | 0.2 | 584 | 0.2 | 220 | 0.2 |
| Molybdenum (Mo) | 19 | 0.0 | 18 | 0.0 | 1 | 0.0 |
| Nickel (Ni) | 60 | 0.0 | 66 | 0.0 | 20 | 0.0 |
| Silicon (Si) | 95,400 | 29.5 | 112,000 | 35.6 | 0 | 0.0 |
| Sodium (Na) | 23,900 | 7.4 | 6,480 | 2.1 | 19,000 | 18.9 |
| Strontium (Sr) | 363 | 0.1 | 378 | 0.1 | 225 | 0.2 |
| Tin (Sn) | 1,230 | 0.4 | 1,300 | 0.4 | 25 | 0.0 |
| Titanium (Ti) | 1,830 | 0.6 | 2,010 | 0.6 | 10 | 0.0 |
| Vanadium (V) | 120 | 0.0 | 140 | 0.0 | 20 | 0.0 |
| Zinc (Zn) | 11,200 | 3.5 | 5,230 | 1.7 | 10,000 | 9.9 |
| **Total** | 323,935 | 100.0 | 314,464 | 100.0 | 100,682 | 100.0 |

It should be noted that the mass of each element presented did not take into account that the insoluble and soluble fractions corresponded to 83 and 17 mass % of the total.
