# Supplementary material for: In vitro toxicoproteomic analysis of A549 human lung epithelial cells exposed to urban air particulate matter and its water-soluble and insoluble fractions
Source: Part Fibre Toxicol. 2017 Oct 2;14:39. doi: 10.1186/s12989-017-0220-6 (PMC5625787; doi:10.1186/s12989-017-0220-6)
Supplement: Supplementary file 2 — Two-way ANOVA results for the A549 protein spots that changed significantly due to particle exposures (n = 3). The SSP number corresponds to the identifier number that PDQuest used to identify the spot based on its coordinate in the gel. The number below Treatment main effect (Trt), Dose main effect (Dose) or interaction between Treatment and Dose (T x D) corresponds to the p-value, where the bolded number emphasized p-value < 0.05. Only the protein spots identified by MALDI-TOF-TOF-MS/MS are provided here ([61]; [62]). The proteins indicated in red (likely degradation product of the native protein) and fold-change indicated in blue (cut-off at ±1.10) were excluded from pathway analysis. Orange colored spots were used for pathway analysis in the 60 μg/cm2 dose. The yellow highlight shows multiple protein spots with the same protein ID. See the Materials and Methods section for more information on the protein spot selection criteria for pathway analysis. Foot Note: § Spot volume intensity normalized to the control (n = 3). †Significant change in protein expression identified by multiple comparison based on Holm-Sidak method (see Materials and Methods), which was used for pathway analysis, and the blank entries imply non-significant changes as compared to the control (i.e., fold-change = 1.0). Those protein spots with p-value <0.05 (based on Two-way ANOVA) but did not pass Holm-Sidak test were excluded. (DOCX 1799 kb) [file 12989_2017_220_MOESM2_ESM.docx]

**
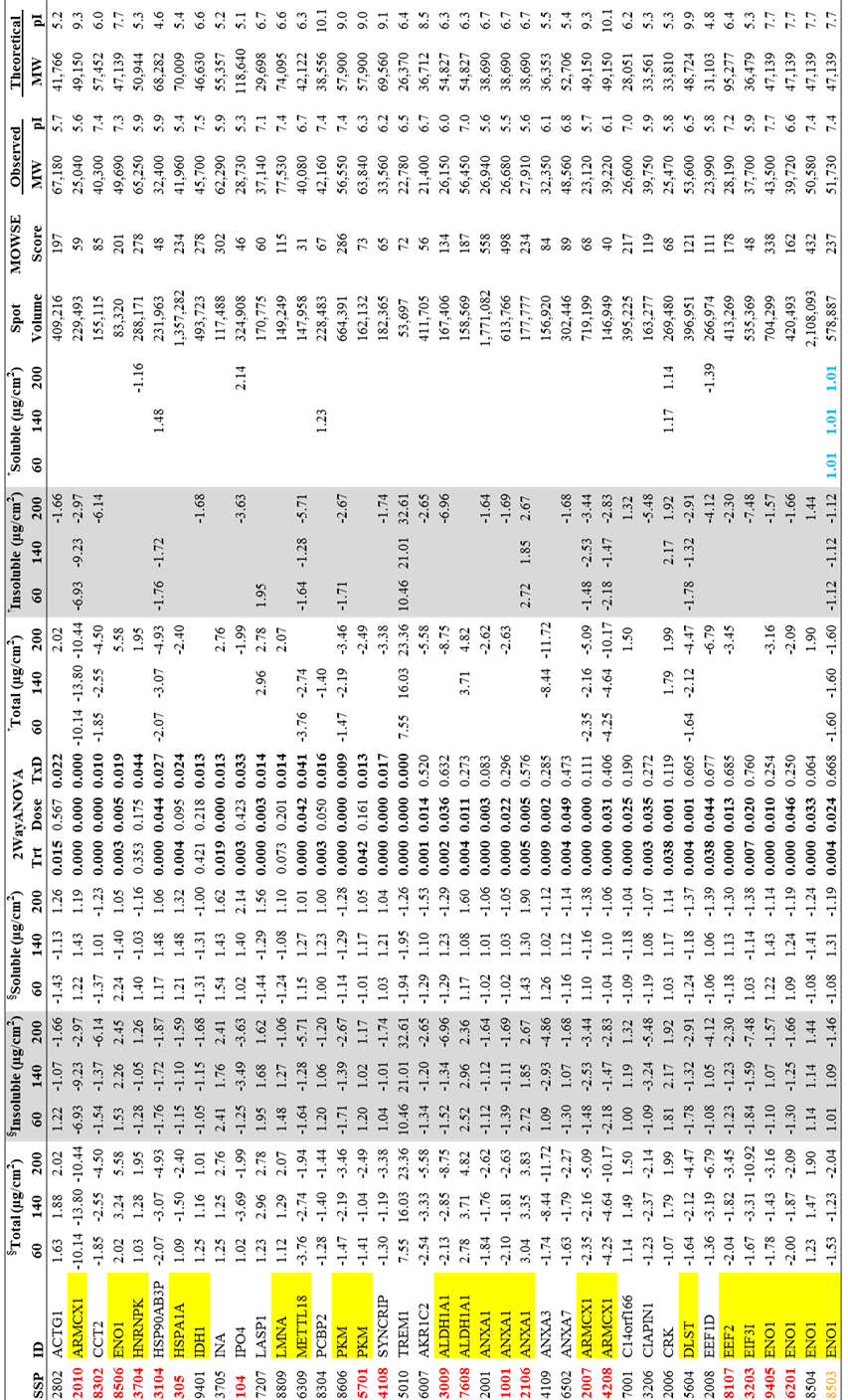
Table S2.** Two-way ANOVA results for the A549 protein spots changed due to particle exposures (n = 3). The SSP number corresponds to the identifier number that PDQuest used to identify the spot based on its coordinate in the gel. The number below *Treatment* main effect (Trt), *Dose* main effect (Dose) or interaction between *Treatment and Dose* (T x D) corresponds to the p-value, where the bolded number emphasized p-value<0.05. Only the protein spots identified by MALDI-TOF-TOF-MS/MS are provided here (Vuong et al., 2016c;Vuong et al., 2016b). The proteins indicated in red (likely degradation product of the native protein) and fold-change indicated in blue (cut-off at ±1.10) were excluded from pathway analysis. Orange colored spots were used for pathway analysis in the 60 μg/cm^2^ dose. The yellow highlight shows multiple protein spots with the same protein ID. See the Materials and Methods section for more information on the protein spot selection criteria for pathway analysis.

**
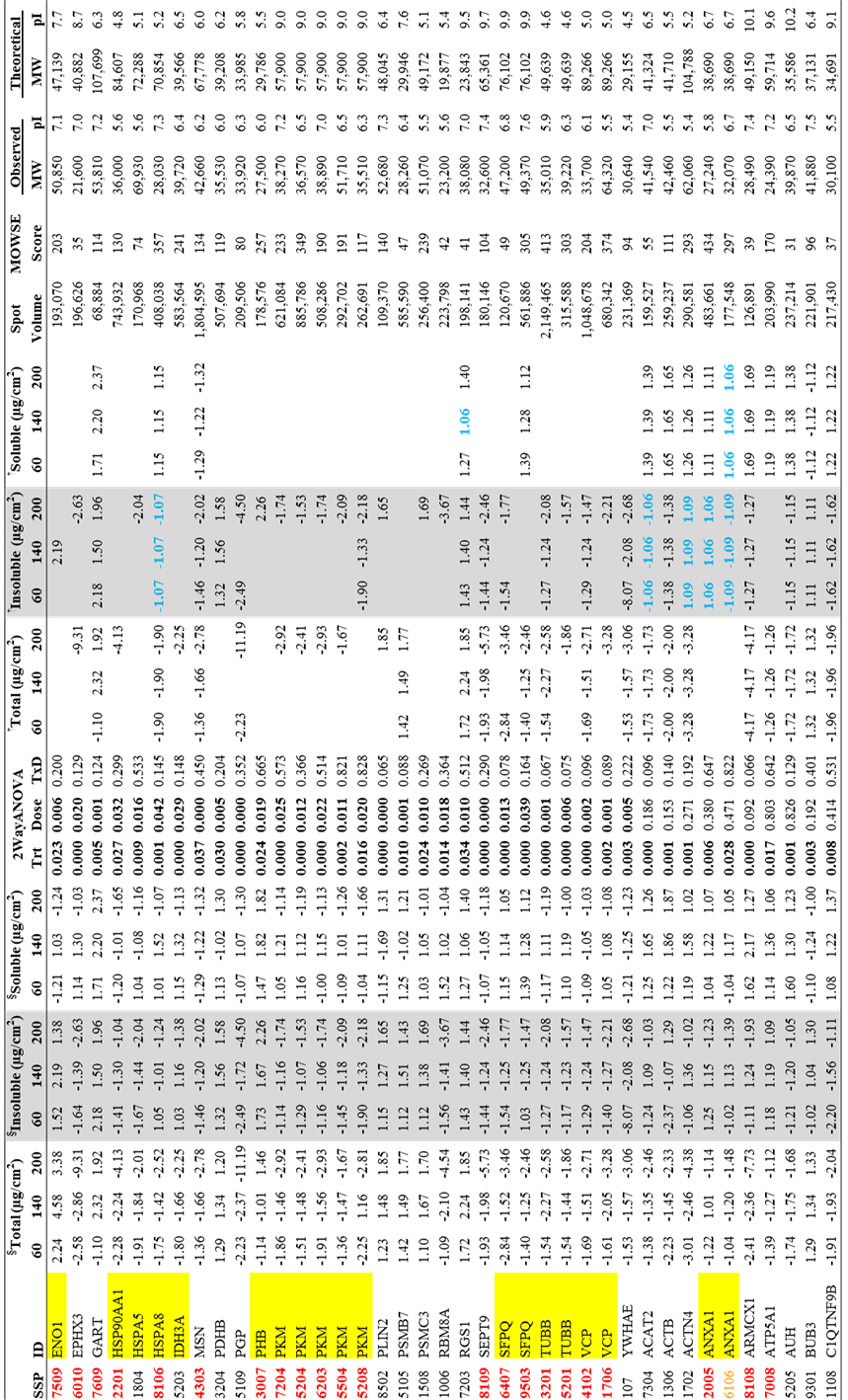
Table S2 (continued…)**

**Table S2 (continued…)**

**
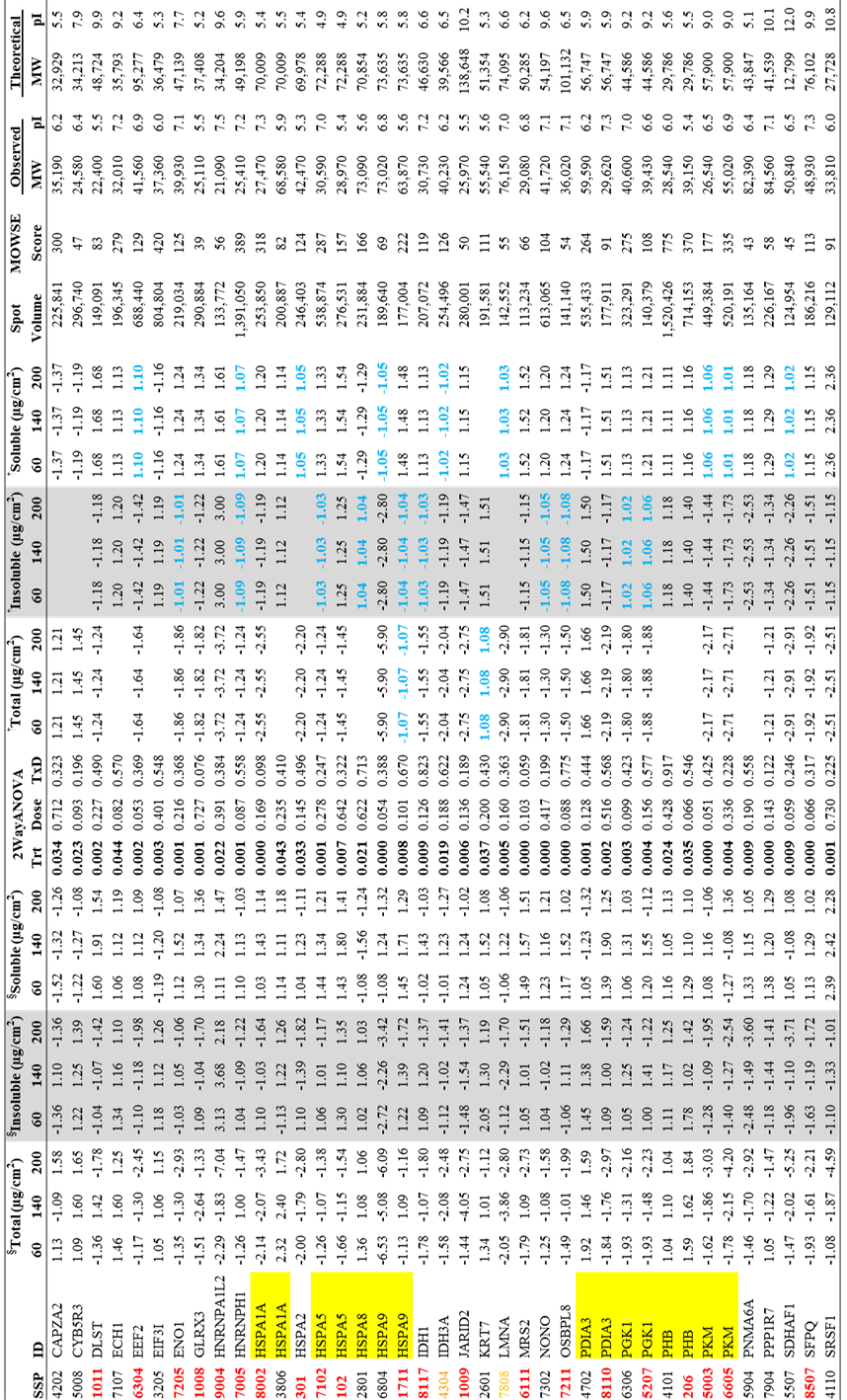
**

**Table S2 (continued…)**

**^
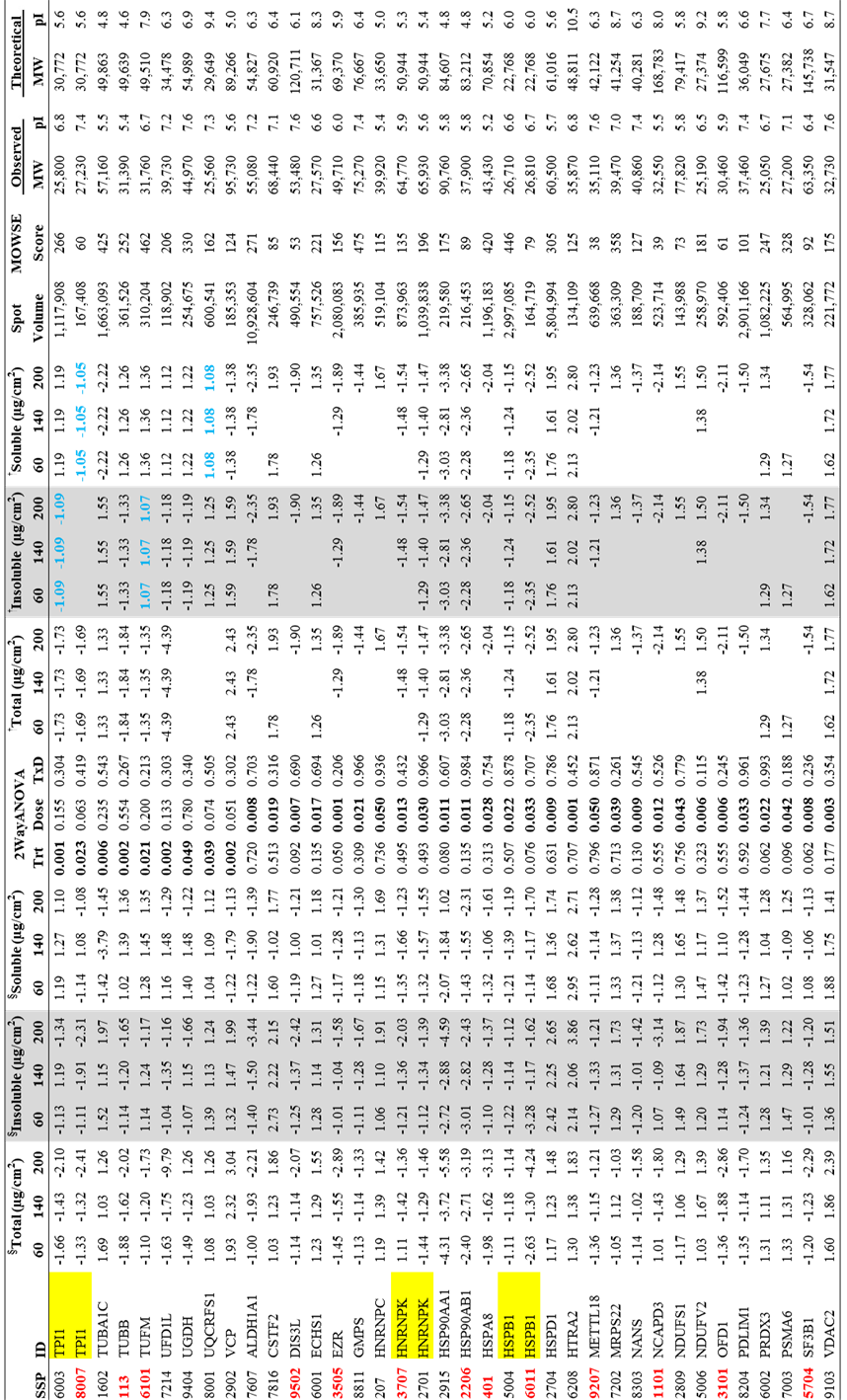
^**

**^§^** Spot volume intensity normalized to the control (n = 3).

**^†^** Significant change in protein expression identified by multiple comparison based on Holm-Sidak method (see Materials and Methods), which was used for pathway analysis, and the blank entries imply non-significant changes as compared to the control (i.e., fold-change = 1.0). Those protein spots with p-value < 0.05 (based on Two-way ANOVA) but did not pass Holm-Sidak test were excluded.
