# Supplementary figures and images for: In vitro toxicoproteomic analysis of A549 human lung epithelial cells exposed to urban air particulate matter and its water-soluble and insoluble fractions
Source: Part Fibre Toxicol. 2017 Oct 2;14:39. doi: 10.1186/s12989-017-0220-6 (PMC5625787; doi:10.1186/s12989-017-0220-6)

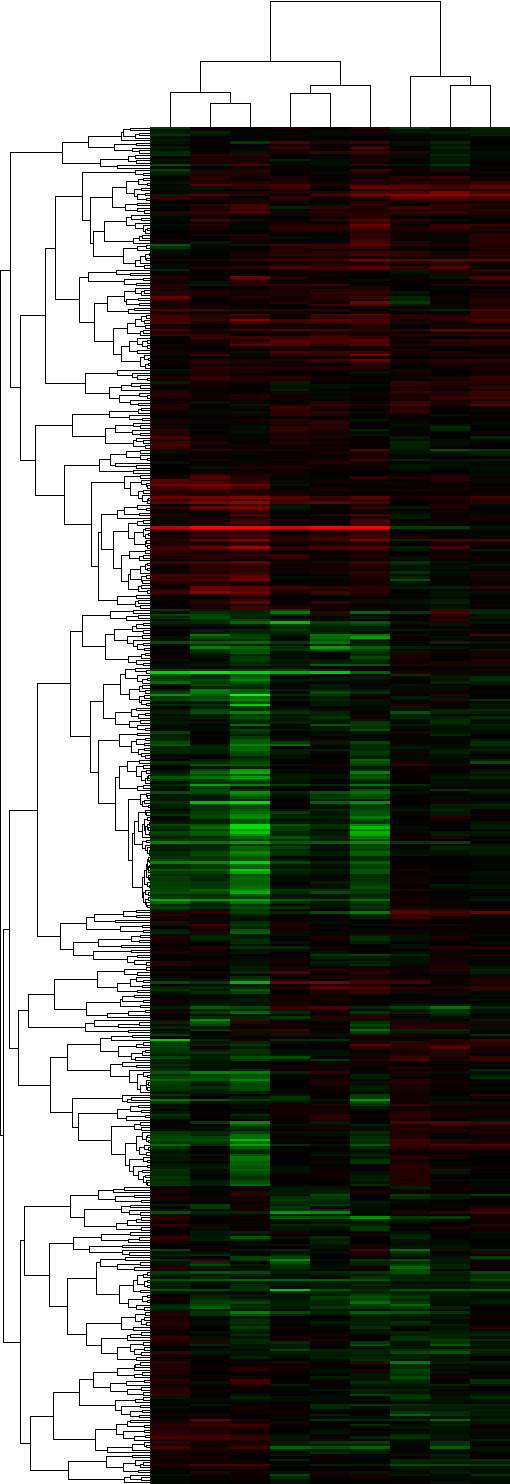


Total: 60

Total: 140

Total: 200

Insoluble: 60

Insoluble: 140

Insoluble: 200

Soluble: 140

Soluble: 60

Soluble: 200


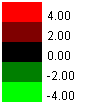


**Figure S1**. Vuong et al., 2017

Supplement: Supplementary file 3 — Unsupervised hierarchical cluster analysis demonstrating the effect of all the tested particles on the proteome of A549 cells. The expressions of all the well-defined protein spots in the 2D gels were examined. The expression of each protein spot was calculated by Log2(Treatment/Control), n = 3. Red is coded for increased expression and green is coded for decreased expression. The number indicates the dose in μg/cm2. (DOCX 196 kb) [file 12989_2017_220_MOESM3_ESM.docx]
