# Supplementary material for: In vitro toxicoproteomic analysis of A549 human lung epithelial cells exposed to urban air particulate matter and its water-soluble and insoluble fractions
Source: Part Fibre Toxicol. 2017 Oct 2;14:39. doi: 10.1186/s12989-017-0220-6 (PMC5625787; doi:10.1186/s12989-017-0220-6)
Supplement: Supplementary file 4 — Top cellular functions in which the proteins in various clusters (in Fig. 3) were involved based on IPA. Only those functions that were significantly (p < 0.05) influenced by more than 5 proteins were presented. (DOCX 12 kb) [file 12989_2017_220_MOESM4_ESM.docx]

Table S3. Top cellular functions in which the proteins in various clusters (in Figure 3) were involved based on IPA. Only those significant (p<0.05) functions that were influenced by more than 5 proteins were presented.

| **Cluster** | **Cellular Function** |
| --- | --- |
| Cluster I | Cellular Movement |
|  | Cellular Growth and Proliferation |
|  | Cell Death and Survival |
|  | Molecular Transport |
|  | Small Molecule Biochemistry |
| Cluster II | *Too few protein available for analysis |
| Cluster III | Cellular Movement |
|  | Carbohydrate Metabolism |
|  | Cellular Growth and Proliferation |
|  | Cell Death and Survival |
|  | Cell Morphology |
| Cluster IV | Lipid Metabolism |
|  | Small Molecule Biochemistry |
|  | Cellular Growth and Proliferation |
| Cluster V | Cell Morphology |
|  | Cellular Function and Maintenance |
|  | Cellular Assembly and Organization |
|  | Cell Death and Survival |
