# Supplementary material for: In vitro toxicoproteomic analysis of A549 human lung epithelial cells exposed to urban air particulate matter and its water-soluble and insoluble fractions
Source: Part Fibre Toxicol. 2017 Oct 2;14:39. doi: 10.1186/s12989-017-0220-6 (PMC5625787; doi:10.1186/s12989-017-0220-6)
Supplement: Supplementary file 5 — Changes in the expression of proteins in various pathways in A549 cells that were exposed to EHC-93 total and its insoluble and soluble fractions (at 60 μg/cm2) examined by hierarchical cluster analysis. These selected pathways were based on the top biological functions identified by Ingenuity Pathway Analysis (in Table 4). The color scales that show fold-changes, Log2(Treatment/Control), were set between −3 to 3 in panels A – C and −2 to 2 in panels D – F. (DOCX 259 kb) [file 12989_2017_220_MOESM5_ESM.docx]

**B) Cell Growth & Proliferation**

**A) Cell Death & Survival**

**C) Cellular Movement**

**E) Acute Inflammation**

**D) Cytoplasm Organization**

**F) ROS Metabolism**


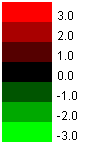


3.0

2.0

1.0

0.0

-1.0

-2.0

-3.0

Total

Insol

Solu


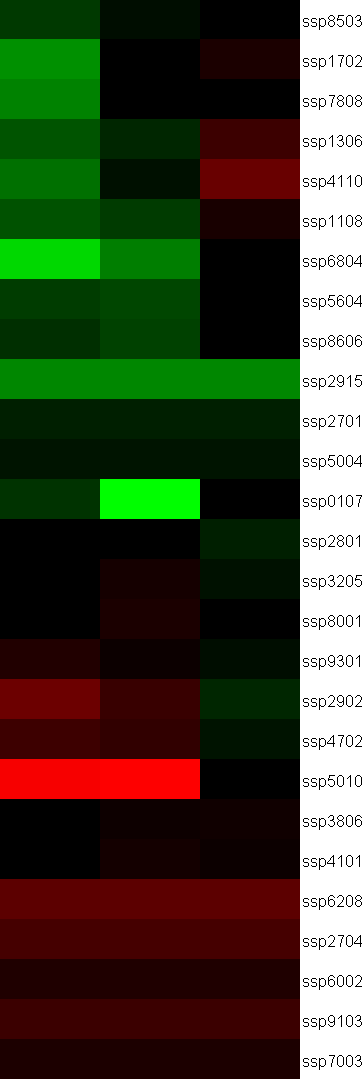

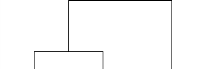

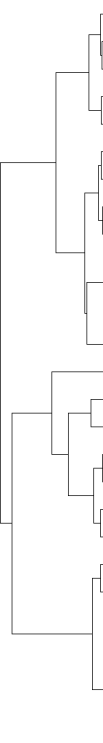


Total

Insol

Solu


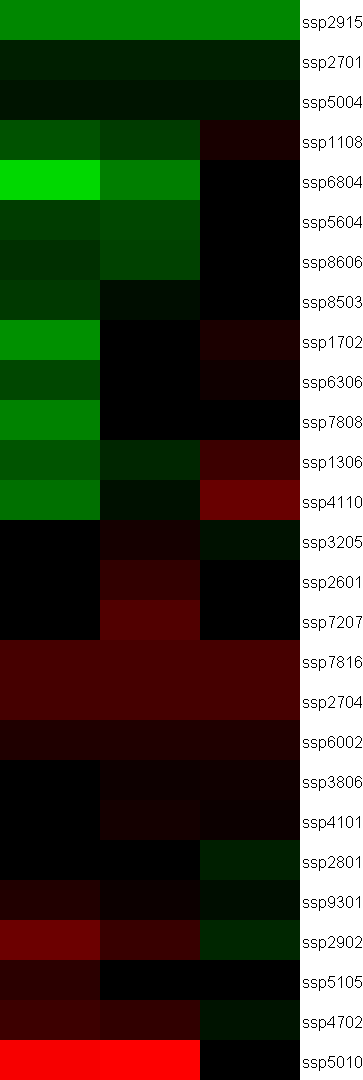

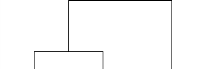

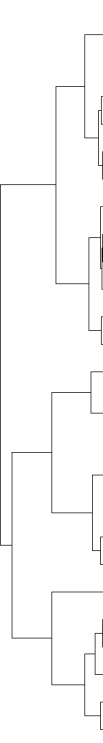

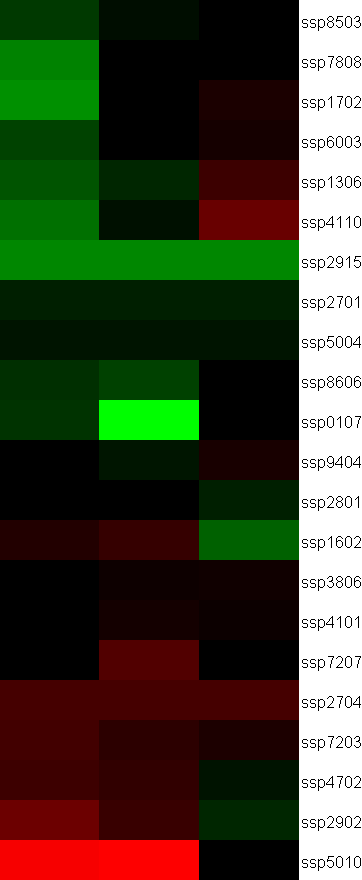


Total

Insol

Solu


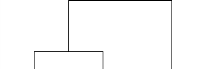

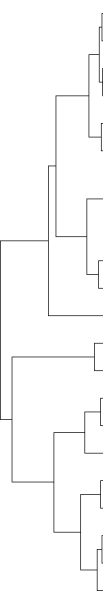

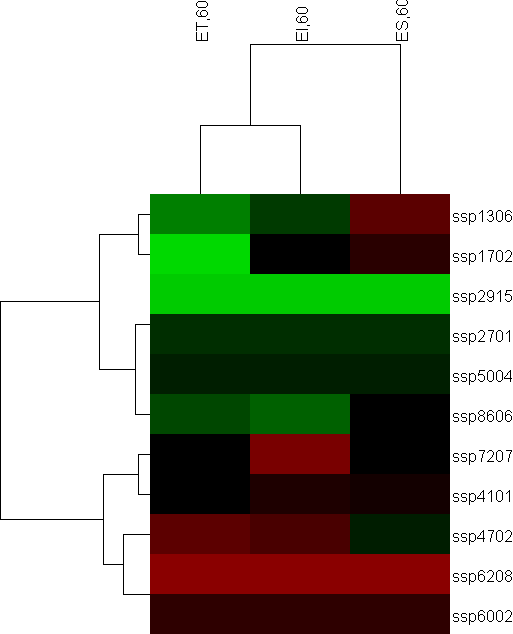

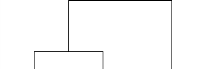


Total

Insol

Solu


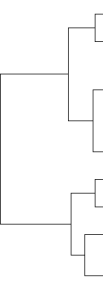

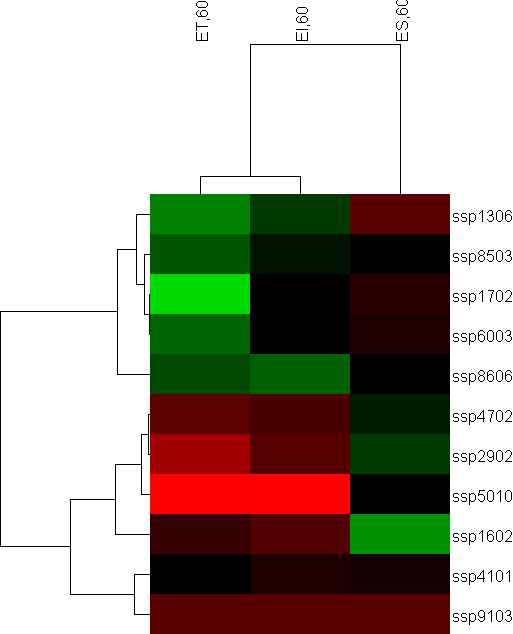

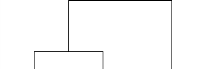


Total

Insol

Solu


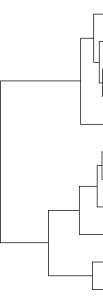

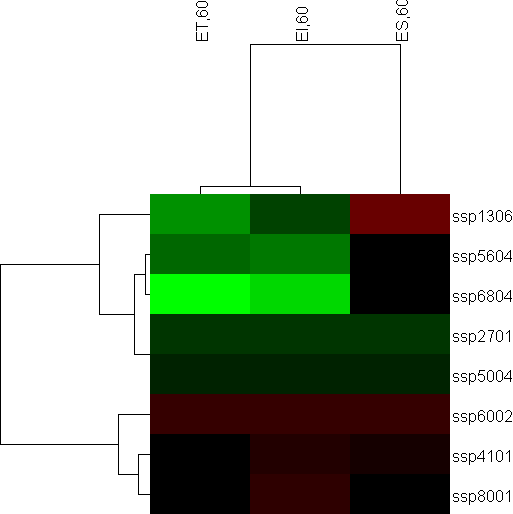

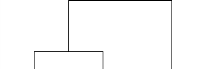

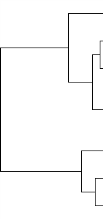


Total

Insol

Solu


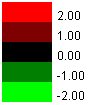


2.0

1.0

0.0

-1.0

-2.0

**Figure S2**. Vuong et al., 2017
